# The metabolic fate of fenclozic acid in chimeric mice with a humanized liver

**DOI:** 10.1007/s00204-018-2274-0

**Published:** 2018-08-09

**Authors:** Anja Ekdahl, Lars Weidolf, Matthew Baginski, Yoshio Morikawa, Richard A. Thompson, Ian D. Wilson

**Affiliations:** 1 MetaSafe AB, Forskargatan 20J, Södertälje, Sweden; 2 DMPK, Cardiovascular, Renal and Metabolism, IMED Biotech Unit, AstraZeneca, Gothenburg, Sweden; 3 Research Planning and Business Development Department, PhoenixBio USA Corporation, 65 Broadway, Suite 605, New York, NY 10006 USA; 4 DMPK, Respiratory, Inflammation and Autoimmunity IMED Biotech Unit, AstraZeneca, Gothenburg, Sweden; 5 Department of Surgery and Cancer, Imperial College, Exhibition Rd, South Kensington, London, SW7 2AZ UK

**Keywords:** Fenclozic acid, Reactive metabolites, Acyl glucuronides, Acyl-CoA conjugates, Hepatotoxicity, Chimeric mouse

## Abstract

The metabolic fate of the human hepatotoxin fenclozic acid ([2-(4-chlorophenyl)-1,3-thiazol-4-yl]acetic acid) (Myalex) was studied in normal and bile-cannulated chimeric mice with a humanized liver, following oral administration of 10 mg/kg. This in vivo animal model was investigated to assess its utility to study “human” metabolism of fenclozic acid, and in particular to explore the formation of electrophilic reactive metabolites (RMs), potentially unique to humans. Metabolism was extensive, particularly involving the carboxylic acid-containing side chain. Metabolism resulted in the formation of a large number of metabolites and involved biotransformation via both oxidative and conjugative routes. The oxidative metabolites detected included a variety of hydroxylations as well as cysteinyl-, *N*-acetylcysteinyl-, and cysteinylglycine metabolites. The latter resulted from the formation of glutathione adducts/conjugates providing evidence for the production of RMs. The production of other classes of RMs included acyl-glucuronides, and the biosynthesis of acyl carnitine, taurine, glutamine, and glycine conjugates via potentially reactive acyl-CoA intermediates was also demonstrated. A number of unique “human” metabolites, e.g., those providing evidence for side-chain extension, were detected in the plasma and excreta of the chimeric liver-humanized mice that were not previously characterised in, e.g., the excreta of rat and C57BL/6 mice. The different pattern of metabolism seen in these chimeric mice with a humanized liver compared to the conventional rodents may offer clues to the factors that contributed to the drug-induced liver injury seen in humans.

**Electronic supplementary material:**

The online version of this article (10.1007/s00204-018-2274-0) contains supplementary material, which is available to authorized users.

## Introduction

Fenclozic acid ([2-(4-chlorophenyl)-1,3-thiazol-4-yl]acetic acid) (Myalex), a potent non-steroidal anti-inflammatory drug (NSAID), discovered in the 1960s, was initially developed as an alternative to the high doses of aspirin used for the treatment of rheumatoid arthritis (Chalmers et al. 1969a, b). Whilst the drug had an excellent safety profile in preclinical species, it was withdrawn from late stage clinical development in patients as a result of hepatotoxicity. The most egregious manifestation of this toxicity was seen in a clinical trial in 12 patients, receiving 400 mg/day of the drug, in which two of the subjects experienced a severe adverse drug reaction (ADR) (Alcock 1970) and further four subjects showed elevated liver enzymes (Hart et al. 1970). Drug-induced liver injury (DILI) was also observed in two subjects at other clinical trial centres as they became jaundiced (Hart et al. 1970). The onset of toxicity in humans on 400 mg/day administration of fenclozic acid was seen between 17 and 35 days after the first dose of the drug and elevated circulating alkaline phosphatase, serum glutamic-oxaloacetic transaminase, and serum glutamic pyruvic transaminase levels were detected (Hart et al. 1970). The biochemical changes seen in the jaundiced subjects were interpreted as being indicative of cholestasis rather than parenchymal cell damage (Hart et al. 1970). All of the patients recovered within a few days of cessation of administration of the drug. A comprehensive search for a suitable preclinical model that recapitulated the DILI seen in patients was unsuccessful (Alcock 1970) and the mechanism for the human-specific DILI remained unresolved. Thus, fenclozic acid presents an interesting problem for the safety assessment of new drug candidates as, in preclinical species, it was not a hepatotoxin but when administered to humans showed itself to result in dose-dependent DILI. As a result, we have explored a range of in vitro systems (Rodrigues et al. 2013) and performed a number of in vivo studies in rodents (Martin et al. 2014; Pickup et al. 2014, 2017; Akingbasote et al. 2017) in efforts to better understand the mechanism(s) underlying the toxicity of fenclozic acid. It was also of interest to see if the methods currently available for evaluating the potential of a new chemical entity (NCE) to cause human toxicity were more able to predict the toxicity of fenclozic acid than those used at the time which the drug was in preclinical development. The in vitro studies (Rodrigues et al. 2013) demonstrated significant levels of time-dependent covalent binding to protein in both NADPH-supplemented liver microsomes and cultured hepatocytes from a range of species including human. Whilst these studies clearly suggested the involvement of oxidative metabolism, attempts at characterizing the metabolites responsible via, e.g., trapping of the reactive metabolites were unsuccessful (Rodrigues et al. 2013). As well as in microsomal incubations, fenclozic acid was assessed in vitro for both P450-dependent and -independent cytotoxicity to THLE cell lines (SV40 T-antigen-immortalized human liver epithelial cells), time-dependent inhibition of important human cytochrome P450 enzymes, inhibition of BSEP (bile salt efflux pump) and MRP2 (human multidrug resistance-associated protein 2), and mitochondrial toxicity to THLE or HepG2 cells, but was without effect (Rodrigues et al. 2013). Support for the hypothesis that P450-based bioactivation and covalent binding to proteins might have a role in the DILI observed with fenclozic acid in humans was provided by a number of in vivo metabolism studies in rodents which, unlike the in vitro investigation, revealed the presence of glutathione-derived metabolites in rat- and mouse-derived samples (Martin et al. 2014; Pickup et al. 2017). In addition, a whole-body autoradiography study using [^14^C]-labelled fenclozic acid in the mouse showed retention of radioactivity in blood, kidney, and liver at 72 h post-dose together with the demonstration of covalent binding to proteins. Despite the observation of acute hepatic necrosis after a single 10 mg/kg oral dose in that study, the repeated oral administration of fenclozic acid to mice at either 50 or 100 mg/kg/day for 7 days did not induce DILI (Akingbasote et al. 2017). If metabolic activation is considered a key initiating factor to DILI (Stepan et al. 2011; Thompson et al. 2016), and assuming that there are unique aspects to the metabolism of the drug in humans, we hypothesized that the use of chimeric mice with a humanized liver (Kamimura et al. 2010; Strom et al. 2010; Scheer and Wilson 2016) might improve the prediction of human liver-based metabolism and toxicity. In the present study, the metabolic fate of fenclozic acid was investigated in the PXB-mouse model, a chimeric mouse with a humanized liver that is highly repopulated with human hepatocytes [chimeric-humanized cDNA-uPA/SCID mice (Ohtsuki et al. 2014)]. In this mouse model, human hepatocytes, infused into immunologically compromised mice, colonize the liver to replace up to 95% of the murine hepatocytes with human hepatocytes. Here, we describe the results of studies undertaken on the metabolic fate of fenclozic acid in the PXB-mouse model.

## Materials and methods

### Chemicals and regents

Fenclozic acid [(2-(4-chlorophenyl)-1, 3-thiazol-4-yl]acetic acid) was obtained from Compound Management (AstraZeneca, Gothenburg, Sweden). Acetonitrile (ACN) LC/MS grade was supplied by Fisher Scientific (Loughborough, UK). Water was supplied from an in-house water purification system (Milli-Q^®^, Merck Millipore, Darmstadt, Germany). Formic acid (FA) was of HPLC grade from ProteoChem (Hurricane, UT, USA).

### Animal dosing

Five male PXB-mice and three male bile-cannulated PXB-mice (12–18 weeks old), with a human hepatocyte replacement index range of 79.7–92.0% (average: 86.5%), were transplanted with human hepatocytes and prepared by PhoenixBio Co., Ltd. Human hepatocytes were obtained from a human donor (Lot BD195) by BD Biosciences (San Jose, CA). The animals were maintained under their standard conditions (e.g., 12 h light/dark cycle with free access to food and water with temperature and humidity controlled). Prior to dosing, the mice were acclimatised for an appropriate period prior to the study start (undertaken at InterVivo Solutions Inc. (IVS) (Mississauga, ON, Canada)) in accordance with the principles of the Animal for Research Act of Ontario and the guidelines of Canadian Council on Animal Care (CCAC) (Guide for the Care and Use of Experimental Animals) and related policies were followed. The protocol was reviewed and approved by the Study Facility’s Institutional Animal Care and Use Committee (IACUC) before the study commenced as per IACUC’s standard operating procedures. For oral dosing, fenclozic acid was dissolved in water at 1 mg/mL administered in a 10 mL/kg dosing volume at a dose of 10 mg/kg, a dose that had been used in the previous studies in the mouse. Two PXB-mice were administered vehicle alone, whilst three intact PXB and three bile-cannulated PXB-mice received the drug. The mice were housed in metabolism cages immediately after dosing. Urine and faeces were collected at – 2 to 0, 0–6, 6–12, and 12–24 h post-dose. Bile was collected from cannulated animals at – 2 to 0, 0–6, 6–12, and 12–24 h. Samples were stored at –80 °C until analysis. Serial blood samples (20, 250 µL at termination) taken from the tail vein were collected into heparin-coated microtubes at 0, 0.25, 0.5, 1, 2, 6, and 24 h post-dose, centrifuged at 3200×*g* for 5 min at 4 °C and plasma transferred into 1.5 mL cryovials. Blood samples were obtained from the control animals at pre-dose and termination. Blood samples were frozen at − 80 °C.

### Sample preparation for metabolite profiling

To obtain metabolite profiles for plasma, individual plasma samples from all time points were used if the sample’s volume was ≥ 15 µL. The plasma was supplemented with three volumes of ACN and vortex mixed to precipitate proteins, followed by centrifugation at 20,000*g* for 10 min at 5 °C. The supernatants were diluted 1:1 with water and injected on to the LC–MS system.

Aliquots (50 or 100 µL) of the pre-dose, 0–6, 6–12, and 12–24 h urine samples were mixed with 5 or 10 µL of ACN, vortexed and centrifuged at 20,000*g* for 10 min at 5 °C. The clear supernatant was taken for LC–MS analysis.

Similarly, aliquots of bile (10 µL) taken from the pre-dose, 0–6, 6–12, and 12–24 h samples were diluted 100 times with 10% ACN in water. Samples were then vortexed and centrifuged at 20,000*g* for 10 min at 5 °C, and the clear supernatant taken for LC–MS analysis.

Aliquots of faeces (ca. 70 mg) from the pre-dose, 0–12, and 12–24 h time points were mixed with 200 µL of water, together with Zirconia beads, and homogenised (10 s, 5,000 rpm) using a Precellys 24 homogenizer (Bertin Instruments, Versailles, France). Then, ACN (500 µL) was added to each sample, and the samples were vortex mixed and centrifuged at 20,000*g* for 10 min at 5 °C. The supernatant was removed and evaporated almost to dryness with N_2_ at room temperature and reconstituted in 200 µL of 30% ACN in water. The samples were centrifuged at 20,000*g* for 10 min at 5 °C and the supernatant analysed by LC–MS.

For the analysis of liver, 200 µL of water was added to ca. 300 mg of tissue, together with Zirconia beads, and the samples were homogenised (10 s, 5,000 rpm) using the Precellys 24 homogenizer. Then, ACN was added (1.0 mL) to each sample, and the samples were vortex mixed and centrifuged at 20,000*g* for 10 min at 5 °C. The supernatant was removed and evaporated almost to dryness with N_2_ at room temperature and then reconstituted in 200 µL of 30% ACN in water. The samples were centrifuged at 20,000*g* for 10 min at 5 °C and the supernatant analysed by LC–MS.

In the case of kidney, the whole right kidney (weight from 100 to 350 mg) was taken, 100 µL of water and Zirconia beads added, and homogenised (10 s, 5,000 rpm) using the Precellys 24 homogenizer. Following homogenisation, 500 µL of ACN was added to each homogenate and the samples were vortex mixed and centrifuged at 20,000*g* for 10 min at 5 °C. The supernatant was removed and evaporated almost to dryness with N_2_ at room temperature and reconstituted in 100 µL of 30% ACN in water. The samples were centrifuged at 20,000*g* for 10 min at 5 °C and the supernatant used in the LC–MS analysis.

### UPLC–MS-based metabolite profiling

Structural characterisation of metabolites and metabolite profiling was performed using an Agilent 1290 Infinity II UHPLC system connected to an Agilent 6560A IM QTOF system (Agilent Technologies, Santa Clara CA, USA) fitted with an electrospray ionisation (ESI) source operated in positive-ion mode over a mass range *m*/*z* 50–1000. Reversed-phase gradient elution was performed on an Acquity U(H)PLC BEH C18 column (2.1 × 100 mm, 1.7 µm, Waters) at room temperature. The mobile phases consisted of 0.1% FA in water (A) and ACN (B). The gradient used was as follows: 10% B at 0 min increased to 70% B at 6.0 min. The solvent composition was held at 70% B for 0.5 min and then decreased to 10% B at 6.7 min. For analysis, 10 µL of each sample was injected. Full-scan MS spectra were obtained over the mass range 50–1000 Da. Targeted MSMS spectra were acquired on selected metabolites at medium isolation width (~ 4 *m*/*z*) using a collision energy of 17 V.

### Metabolite identification by MS

Accurate masses of metabolites were determined from the protonated and/or sodiated molecules in the positive ESI–TOF–MS mode. A mass window of ± 2 Da was used for the precursor ion to retain the Cl-35/Cl-37 isotope clusters in MSMS product ion mode and accurate masses of fragment ions were obtained following collision-induced dissociation. The major fragmentation pathways seen in the mass spectrum of fenclozic acid (see Online Resource 1, Figs. S1 and S2) were elucidated and used as the basis for the interpretation of those of the metabolites detected in samples and to propose structures. The mass error for each proposed metabolite and fragment ion structure was < 5 ppm (range, e.g., 0.03–1.27 mDa for metabolites seen in plasma) compared to the theoretical exact mass. For those metabolites where no MSMS data could be acquired, identification was based on the accurate mass of the protonated and/or sodiated molecules and reference to previously published work from our group (Martin et al. 2014 and; Pickup et al. 2017).

## Results

As indicated in the experimental section, following oral administration of fenclozic acid to PXB-mice at 10 mg/kg, to both intact and bile duct cannulated animals, metabolite profiles were determined by UHPLC–MS for plasma, bile, and urine, as well as in extracts of liver, kidney, and faeces [as described for previous studies in the C57BL/6 mouse (Pickup et al. 2017)]. The metabolites detected in the various samples are listed in Table 1 (further mass spectral data and mass spectra supporting the assignment of structures are provided in Online Resource 1, Table S1 and Figs. S8–S30) using the numbering system employed in Martin et al. (2014) and Pickup et al. (2017), with additions to take into account the novel metabolites (M25–M37) detected in the present study. To facilitate inter-species, inter-strain, and inter-model comparison, this table also lists the metabolites observed in recent rodent studies (Martin et al. 2014; Pickup et al. 2017).

**Table 1:** Summary of fenclozic acid metabolites detected in PXB-mice and recent rodent studies

	PXB-mouse						C57BL/6 mouse		HRN-mouse		Rat	
	Plasma	Urine	Bile	Faeces	Liver	Kidney	Urine	Bile	Urine	Faeces	Urine	Bile
Fenclozic acid	++^a^	++	++	++	++	++	D^c^	D	D	D	D	D
M1-M5	++(one)^b^	++ (one)			++ (one)	++(one)		M2, M3			M1-M5	M1-M3
M6 or M7		++						M6			M6, M7	M6, M7
M8	+	+				+	D				D	D
M9		+	+				D					D
M10	++	++	++		++	++	D				D	D
M11	+++	+++	+++		+++	+++		D	D		D	D
M12	++	++	++		++	++	D					D
M13	++	++	++	++	++	++	D	D	D	D	D	D
M14	++	++	++	++	++	++	D	D	D	D		D
M15											D	D
M16							D	D				D
M17		++										D
M18								D				D
M20								D				
M21								D				
M22							D	D				
M23								D				
M24	+	+						D				
M25	++	++										
M26	+++	+++			+++	+++						
M27	+											
M28	+	+		+								
M29 ^d^	+	+				+						
M30		+				+						
M31		+										
M32						+						
M33	+++				+++	+++						
M34	+++		+++		+++	+++						
M35	++	++			++	++						
M36 ^e^	+											
M37	++				++	++						

Fenclozic acid and metabolites detected in the plasma, urine, bile, faeces, and liver and kidney extracts of rodents, including chimeric PXB-mice

The metabolite enumeration is adapted from Martin et al. (2014) and Pickup et al. (2017) where applicable. Metabolites M25–M37 were only seen in the PXB-mice (supporting mass spectra provided in supplementary data Figs. S8–S30)

^a^+++, ++, + signify high, medium, and low abundance, respectively, as given by LC–MS integrated peak areas

^b^“(one)” indicates that one out of five previously reported (M1–M5) hydroxylated metabolites was detected

^c^D is “detected” in referenced studies. Data from the previous studies included in the table are retrieved from Pickup et al. (2017) (C57BL/6 Mouse), Pickup et al. (2014) (HRN Mice), and Martin et al. (2014) (Rat)

^d^M29 corresponds to either metabolite M20, M22, or M23 in Pickup et al. (2017)

^e^In all but one plasma sample

### Plasma metabolite profiles

**Fig. 1:**
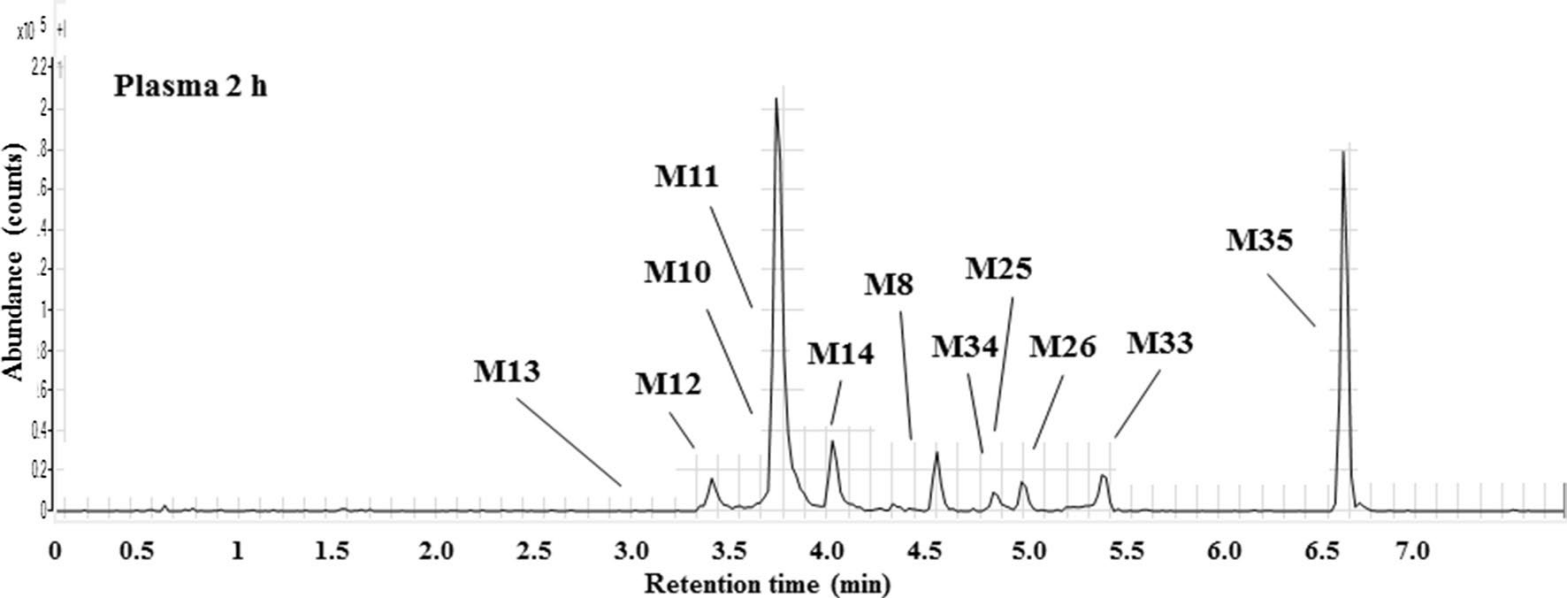
Summed extracted ion chromatogram showing a selection of metabolites detected in plasma at 2 h post-dose. The metabolites were selected, because they were most abundant in this sample and because they represent conjugative metabolism, chain elongation, and the decarboxylation pathways. The chromatogram does not include fenclozic acid (retention time 4.4 min)

Plasma extracts obtained from PXB-mice were profiled for the presence of fenclozic acid and circulating drug-related material using qualitative LC–MS. In contrast to the C57BL/6 mouse where only unchanged fenclozic acid and traces of the glycine conjugate (M14) were detected (Pickup et al. 2017), the metabolite profile obtained for plasma 2 h after oral administration of fenclozic acid to liver-humanized PXB-mice was complex, and contained a wide range of metabolites (see Fig. 1) (a similar profile was obtained for the 6 h sample, data not shown). It should be noted that this figure shows only the signal intensity of the metabolites, without taking into account any potential changes in MS response caused by the structural modifications resulting from biotransformation. In terms of intensity, the major circulating component was the unchanged drug itself, which is not shown in the mass chromatogram provided in Fig. 1 as the signal for it would have dwarfed those of the metabolites.

**Fig. 2:**
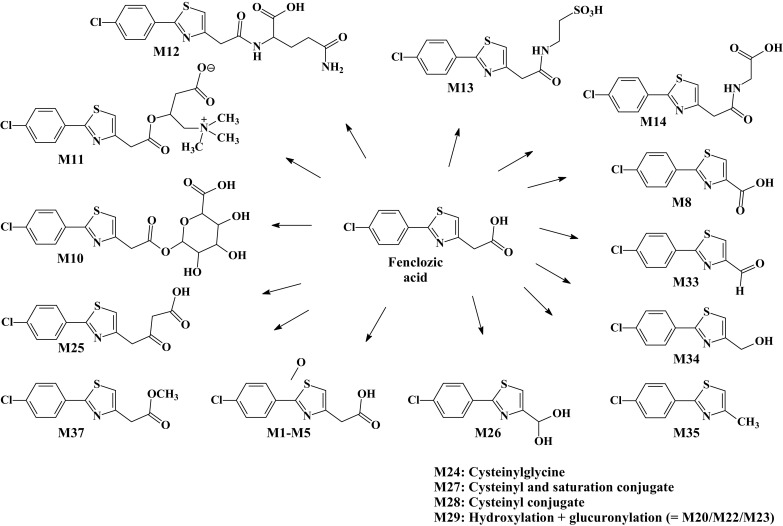
Fenclozic acid and metabolites detected in the plasma of chimeric-humanized PXB-mice (M29 corresponds to either metabolite M20, M22, or M23 in Pickup et al. (2017)

The circulating drug-related material seen in the plasma from both intact and bile duct-cannulated PXB-mice comprised unchanged drug as well as both oxidised and conjugated metabolites. The structures of the fenclozic acid-derived metabolites, including hydroxylated (M1–M5) and side-chain-shortened (M8) compounds, as well as acyl carnitine (M11), glutamyl (M12), taurine (M13), glycine (M14), and glucuronide conjugates (M10) found in plasma are illustrated in Fig. 2.

**Fig. 3:**
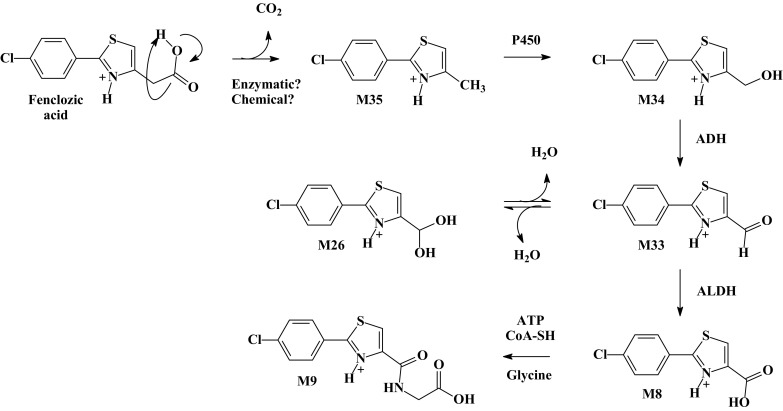
Proposed decarboxylation pathway for fenclozic acid via M35, M34, M33, and M26, leading to the formation of M8 and thence to M9 via glycine conjugation

The unusual decarboxylated compound M35 was originally reported by Foulkes (1970) as “metabolite V”. Whilst Foulkes demonstrated that, by dosing the [^14^C]-carboxyl labelled isotopolog of fenclozic acid and monitoring of exhaled ^14^CO_2_, “metabolite V” could form in vivo it was also shown that it could arise via the chemical decomposition of fenclozic acid in neutral phosphate buffer. It is, therefore, unclear whether the presence of this compound results solely from the biotransformation of fenclozic acid or its degradation, or a combination of both. However, it is, perhaps, noteworthy in this context that this compound was not observed in any of the recently conducted in vitro (Rodrigues et al. 2013) or rodent in vivo studies (Pickup et al. 2014, 2017; Martin et al. 2014). Once formed, M35 provides the basis for a logical progression of reactions that results in the detection of a number of novel metabolites M26, M33, M34, M8, and M9 (see Fig. 3) in the circulation.

As Fig. 3 indicates, it is likely that M26 and M33 represent an equilibrium mixture between the aldehyde (M33) and its hydrate (M26) as recently demonstrated for (1-[(2*R*)-2-[[4-[3-chloro-4-(2-pyridyloxy)anilino]quinazolin-5-yl]oxymethyl]-1-piperidyl]-2-hydroxy) following incubation with human liver microsomes (Martin et al. 2017).

The biotransformation of fenclozic acid to M33, M34, and M35 by microorganisms has also been reported (Howe et al. 1972).

Another potential route for the formation of the carboxylic acid M8 is via CYP P450-mediated oxidative decarboxylation of fenclozic acid of the type described by Grillo et al. (2008), providing a novel bioactivation reaction for diclofenac.

In addition to these compounds, the presence of a metabolite with a mass spectrum consistent with side-chain extension (M25) was also detected.

These data for plasma are summarized in Table 1.

### Metabolite profiles for bile, liver, and faecal extracts

Extracts of liver samples obtained for the bile duct-cannulated PXB-mice provided a metabolite profile that contained many of the metabolites detected in plasma. The bile samples were also similar in content lacking only M1–M5, M26, M33, M35, and M37, but also containing the glycine conjugate of the side-chain-shortened fenclozic acid M9 and the glutathione-derived cysteinylglycine conjugate M24. Faecal extracts contained fenclozic acid and the glycine and (in one sample only) taurine conjugates (M13 and M14, respectively). These data are summarized in Table 1 and illustrated in Online Resource 1, Figs. S3–S5 for bile, liver, and faecal extracts, respectively.

### Metabolite profiles for kidney extracts and urine

As with liver, there was a close correspondence between the metabolites of fenclozic acid detected in extracts of the kidney and those present in plasma. Indeed, the only metabolite not present in plasma that was detected in kidney was M32, an oxygenated metabolite that was also conjugated with glutathione. The metabolites seen in urine also showed considerable similarity to those in the kidney extracts with only M33, M34, and M37 absent. Uniquely present in urine was the oxygen containing N-acetylcysteinyl conjugate M17, presumably derived from the corresponding glutathione conjugate M32. These data are summarized in Table 1 and illustrated in Figs. S6 and S7 for kidney extracts and urine, respectively.

## Discussion

The extensive biotransformation of fenclozic acid in chimeric liver-humanized PXB-mice resulted overall in a complex mixture of metabolites formed via both functionalization and conjugation. As is clear from Table 1, a number of these metabolites were previously detected in both Han Wistar rat and C57BL/6 mouse studies (M1–14, M17) (Martin et al. 2014; Pickup et al. 2017), whilst others were seen only in either the C57BL/6 mouse (M16, M18, M20–M24) or the Han Wistar rat (M15). Among the metabolites detected in this present study were a number of glutathione-derived conjugates (M17, M24, M27, M28, M30–M32) suggesting the formation of electrophilic intermediary metabolites deactivated via glutathione conjugation. A number of these glutathione-derived conjugates were PXB-mouse-specific (M27, M28, M30, and M31). Evidence for metabolic bioactivation during the metabolism of fenclozic acid has been indicated by the detection of high covalent binding in vitro (Rodrigues et al. 2013) and also in vivo in the mouse (Pickup et al. 2017) as well as the identification of a number of glutathione-derived metabolites in both mouse (M16 and M18) (Pickup et al. 2017) and rat (M16–M18) (Martin et al. 2014). In addition to the production of RMs, as indicated by these several glutathione-derived metabolites, a large number of acyl conjugates were identified (M11–M14). This seemingly diverse collection of conjugated metabolites of fenclozic acid all have in common their formation via a route involving ATP activation and CoA conjugation via acyl-CoA synthases (Darnell and Weidolf 2013). The reactivity of acyl-CoA conjugates has been widely studied, although not as thoroughly as that of acyl-glucuronide conjugates of carboxylic acid drugs. Nonetheless, acyl-CoA conjugates have been shown to be 40–70 times more reactive towards glutathione, as a surrogate for nucleophilic biomacromolecules, than the corresponding acyl-glucuronide (Darnell and Weidolf 2013, and references cited therein). The direct reactivity toward biomacromolecules by fenclozic acid metabolites formed in human liver microsomes, supplemented with either UDPGA or ATP and CoA, was assessed as measured by covalent binding (Darnell et al. 2015). In that study, covalent binding of the acyl glucuronide of fenclozic acid was not different from the non-cofactor supplemented incubation, whereas covalent binding of the acyl-CoA conjugate was 5.5-fold higher than its non-supplemented control incubation, thus indicating a significant difference in reactivity between the two types of conjugate. The toxicological potential of the CoA-conjugated acids can be explained by spontaneous transacylation of the aglycone to nucleophilic sites on proteins and other biomacromolecules, thereby modifying the proteins’ function, interfering with cell signalling or other essential processes. Another concern for high-dose carboxylic acid drugs is that they may deplete cells of CoA and, again, interfere with endogenous lipid metabolism (Darnell and Weidolf 2013). Considering the large variety of metabolites formed via CoA conjugation, it is evident that fenclozic acid has the potential to significantly interfere with fatty acid β-oxidation as well as fatty acid synthesis. Of particular toxicological concern is that of carnitine depletion caused by exogenous carboxylic acids such as, e.g., pivalic acid, a metabolite of the antibiotic prodrug pivampicillin. The pivaloyl-carnitine conjugate effectively removes carnitine from the important transportation of fatty acids from the cytosol into the mitochondrion, thereby causing impaired energy production, potentially resulting in cell death (Ito 2009). Whether the carnitine conjugate of fenclozic acid (M11) is formed to such an extent that it could cause these effects remains to be further examined.

As well as conjugation, there was evidence of considerable metabolism in relation to the carboxylic acid-containing side chain. Thus, side-chain shortening resulted in the formation of a series of related metabolites (as shown in Fig. 3) including the methyl- (M35), hydroxymethyl- (M34), aldehyde- (M33), aldehydehydrate- (M26) and carboxylic acid (M8) metabolites of the drug, as well as the glycine conjugate (M9) produced from M8 as formed via CoA conjugation.

**Fig. 4:**
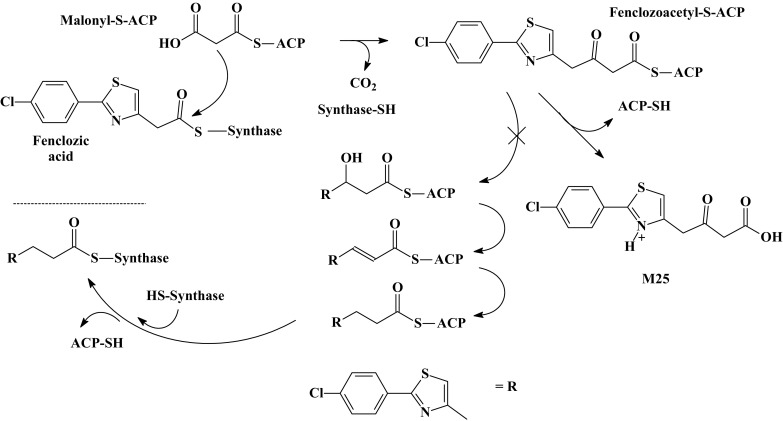
Proposed scheme for the side-chain elongation route leading to the formation of M25 from fenclozic acid following conjugation to CoA and entry into the malonyl-CoA fatty acid biosynthetic pathway with addition of one C2 unit to the carboxylic acid-containing side chain (malonyl-S-ACP: malonyl-(acyl-carrier-protein) carboxy-lyase)

Clearly, whilst it is tempting to claim some of these metabolites as being uniquely human, M26 and M33–M35 form part of an obvious metabolic sequence leading to M8, so that both the rat and mouse, which also produce M8, must also be exposed to these compounds to some extent. However, systemic exposure to M26, M33, and M35 was much greater in the chimeric-humanized liver PXB-mouse compared to the C57BL/6 mouse. In addition to these side-chain-shortened metabolites, the chimeric-humanized liver PXB-mouse produced a truly unique metabolite, M25, the mass spectral data for which was consistent with a biotransformation resulting in side-chain elongation (Fig. 4). The formation of metabolite M25 may be explained by CoA conjugation of fenclozic acid and entry into the malonyl-CoA fatty acid synthesis pathway, resulting in a chain elongation of one C2 unit (see Fig. 4). Such side-chain-elongated metabolites have been described previously for several carboxylic acids, e.g., cyclopropanoic acid for which up to eight C2 units have been added, or phenylacetic acid forming long-chain *ω*-phenyl acids (Dodds 1995).

As highlighted in Table 1, there are numerous similarities between the metabolic fate of fenclozic acid in rodents, such as the rat and mouse, and the chimeric liver-humanized PXB- mice studied here. The detection of glutathione-derived conjugates in an in vivo human-like system in the current study may provide further support for a mechanism of toxicity in humans based, at least in part, on the formation of reactive metabolites as suggested by the previous in vitro (Rodrigues et al. 2013) and in vivo rodent (Martin et al. 2014; Pickup et al. 2017) studies. However, the quantities of the glutathione-derived metabolites detected in the present study do not appear to have been present in disproportionate amounts compared to these previous rodent studies. Consequently, our attention is drawn to the apparent differences in the biotransformation of fenclozic acid observed between chimeric mice with humanized livers and non-humanized rodent models, e.g., the intermediate metabolites of the decarboxylation pathway (M26 and M33–M35; Fig. 3), the clearly unique CoA-mediated side-chain-elongated metabolite (M25; Fig. 4) as well as the previously not seen metabolites related to glutathione conjugation of RMs of fenclozic acid (M27, M28, M30 and M31; Online Resource 1, Table S1). Additional studies with radiolabelled fenclozic acid would be required to make a quantitative comparison to determine if levels of in vivo covalent binding to tissue proteins, etc., were higher in PXB-mice compared to non-humanized mice. However, based on in vitro screening studies examining the hepatotoxicity of a range of drugs (Thompson et al. 2012), we believe that, in many cases, DILI may result from the simultaneous involvement of a number of different mechanisms. Given that the changes seen in patients that led to the withdrawal of the drug from development were considered to be indicative of cholestasis rather than parenchymal cell damage (Hart et al. 1970), it may be well that the observed DILI was not due only to the formation of reactive metabolites. The combination of RM production and metabolites with the potential for disruption of lipid and energy metabolism as a possible explanation for the observed human toxicity of fenclozic acid is beguiling and warrants further exploration.

In conclusion, this study shows that the chimeric-humanized PXB-mouse can potentially be a useful in vivo model of human metabolism via the discovery of novel metabolites of fenclozic acid that have not previously been seen in rodents or in vitro studies with human hepatocytes. The limitation of the study is that, as the drug was withdrawn from development some decades ago, there are no recent human studies on the metabolism of fenclozic acid with which to compare our data. Other reports indicate that, while the model is quite successful for some compounds, it fails for others, a drawback that needs further understanding (Kamimura et al. 2010). One also has to consider the variability in CYP and transporter expression in the transplanted hepatocytes that can be quite considerable and that these depend on the donor genotype and phenotype (Ohtsuki et al. 2014). The chimeric-humanized mouse model is promising, but would benefit from more thorough characterization to explore its scopes and limitations for reliable prediction of human in vivo drug metabolism (Strom et al. 2010). Nevertheless, the unusual metabolites of fenclozic acid seen, resulting from the biotransformation of the carboxylic acid-containing side chain in particular, suggest a number of possible avenues of further investigation which we will explore in further studies.

## Electronic supplementary material

Below is the link to the electronic supplementary material.

Supplementary material 1 (PDF 739 KB)
