# Supplementary material for: The metabolic fate of fenclozic acid in chimeric mice with a humanized liver
Source: Arch Toxicol. 2018 Aug 9;92(9):2819–28. doi: 10.1007/s00204-018-2274-0 (PMC6132692; doi:10.1007/s00204-018-2274-0)

Archives of Toxicology

The Metabolic Fate of Fenclozic Acid in Chimeric Mice with a Humanized Liver

Anja Ekdahl[1], Lars Weidolf[2*], Mathew Baginski[3], Yoshio Morikawa[3], Richard A.Thompson[4], Ian D Wilson[5*]

[1]MetaSafe AB, Forskargatan 20J, Södertälje, Sweden

[2]DMPK, Cardiovascular, Renal and Metabolism, IMED Biotech Unit, AstraZeneca, Gothenburg, Sweden

[3]Research Planning & Business Development Department, PhoenixBio USA Corporation, 65 Broadway, Suite 605, New York, NY 10006, USA

[4]DMPK, Respiratory, Inflammation & Autoimmunity IMED Biotech Unit, AstraZeneca, Gothenburg, Sweden

[5]Dept. of Surgery and Cancer, Imperial College, Exhibition Rd, South Kensington, London SW7 2AZ, UK

Corresponding authors: Ian D Wilson (i.wilson@imperial.ac.uk) and
Lars Weidolf (lars.weidolf@astrazeneca.com)

Online Resource 1

Table S1 MS data for found metabolites of fenclozic acid

Figure S1-S2 Mass spectrum and fragmentation pathways of fenclozic acid

Figure S3-S7 Metabolite schemes of fenclozic acid in excreta and tissue extracts

Figure S8-S30 Mass spectra and structures of metabolites of fenclozic acid

Table S1 Detected protonated metabolites of fenclozic acid (FA). Bolded metabolites are those included in Fig.1 of the main text showing extracted ion chromatogram

**Compound name**	**MH^+**	**$M+Na^+$**	**Acc Mass (MH^+)**	**Exact Mass (MH^+)**	**mDa**	**ppm**	**Composition**	**Descriptor**
Fenclozic acid	yes		254.0033	254.004253	-1	-3.9	C11H9ClNO2S	FA
M1-M5	yes		269.999	269.999168	-0.2	-0.7	C11H9ClNO3S	+ O
M6	yes		272.0142	272.014818	-0.6	-2.2	C11H11ClNO3S	+ H_2O
M7	yes		-”-	-”-			-”-	-”-
**M8**	**yes**	**yes**	**239.9875**	**239.988603**	**-1.1**	**-4.6**	**C10H7ClNO2S**	**Decarb to acid**
M9	yes		297.0098	297.010067	-0.3	-1	C12H10ClN2O3S	Decarb to acyl glycine
**M10**	**yes**	**yes**	**430.0356**	**430.036341**	**-0.7**	**-1.6**	**C17H17ClNO8S**	**+ Gluc Acid**
**M11**	**yes**		**397.0988**	**397.098882**	**-0.1**	**-0.3**	**C18H22ClN2O4S**	**+ Carnitine**
**M12**	**yes**	**yes**	**382.062**	**382.062831**	**-0.8**	**-2.1**	**C16H17ClN3O4S**	**+ Glutamine**
**M13**	**yes**	**yes**	**361.0071**	**361.008353**	**-1.1**	**-3.6**	**C13H14ClN2O4S2**	**+ Taurine**
**M14**	**yes**	**yes**	**311.0251**	**311.025717**	**-0.6**	**-1.9**	**C13H12ClN2O3S**	**+ Glycine**
M17	yes		433.0293	433.029483	-0.2	-0.5	C16H18ClN2O6S2	+ O + NAc-Cys-SH
M24	yes	yes	430.0297	430.029817	-0.1	-0.2	C16H17ClN3O5S2	+ HS-CysGly – 2 H
**M25**	**yes**	**yes**	**296.0145**	**296.014818**	**-0.3**	**-1**	**C13H11ClNO3S**	**+ C2 elongation**
**M26**	**yes**	**yes**	**242.0036**	**242.004253**	**-0.7**	**-2.9**	**C10H9ClNO2S**	**Decarb to aldehyde + H2O**
M27	yes		375.0229	375.024003	-1.1	-2.9	C14H16ClN2O4S2	+ Cys-SH
M28	yes		373.0088	373.008353	0.4	1.1	C14H14ClN2O4S2	+ Cys-SH – 2 H
M29 (M20/M22/M23)	yes	yes	446.0308	446.031256	-0.5	-1.1	C17H17ClNO9S	+ O + Gluc Acid
M30	yes		417.0314	417.034568	-3.2	-7.8	C16H18ClN2O5S2	+ NAc-Cys-SH
M31	yes		415.0183	415.018918	-0.6	-1.4	C16H16ClN2O5S2	+ NAc-Cys-SH – 2 H
M32	yes		575.0665	575.067325	-0.8	-1.4	C21H24ClN4O9S2	+ GSH + O – 2 H
**M33**	**yes**	**yes**	**223.9936**	**223.993688**	**-0.1**	**-0.4**	**C10H7ClNOS**	**Decarb to aldehyde**
**M34**	**yes**		**226.0095**	**226.009339**	**0.2**	**0.9**	**C10H9ClNOS**	**Decarb to alcohol**
**M35**	**yes**		**210.014**	**210.014424**	**-0.4**	**-1.9**	**C10H9ClNS**	**Decarb to methyl**
M36	yes	yes	323.9692 (Na+)	323.970942	-1.7	-5.2	C11H8ClNO5S Na+	FA + 3 O
M37	yes		268.0196	268.019903	-0.3	-1.1	C12H11ClNO2S	+ CH2

Figure S1. Q-ToF MSMS product ion spectrum of fenclozic acid (FAH^+), narrow isolation width @ 1.3 Da.

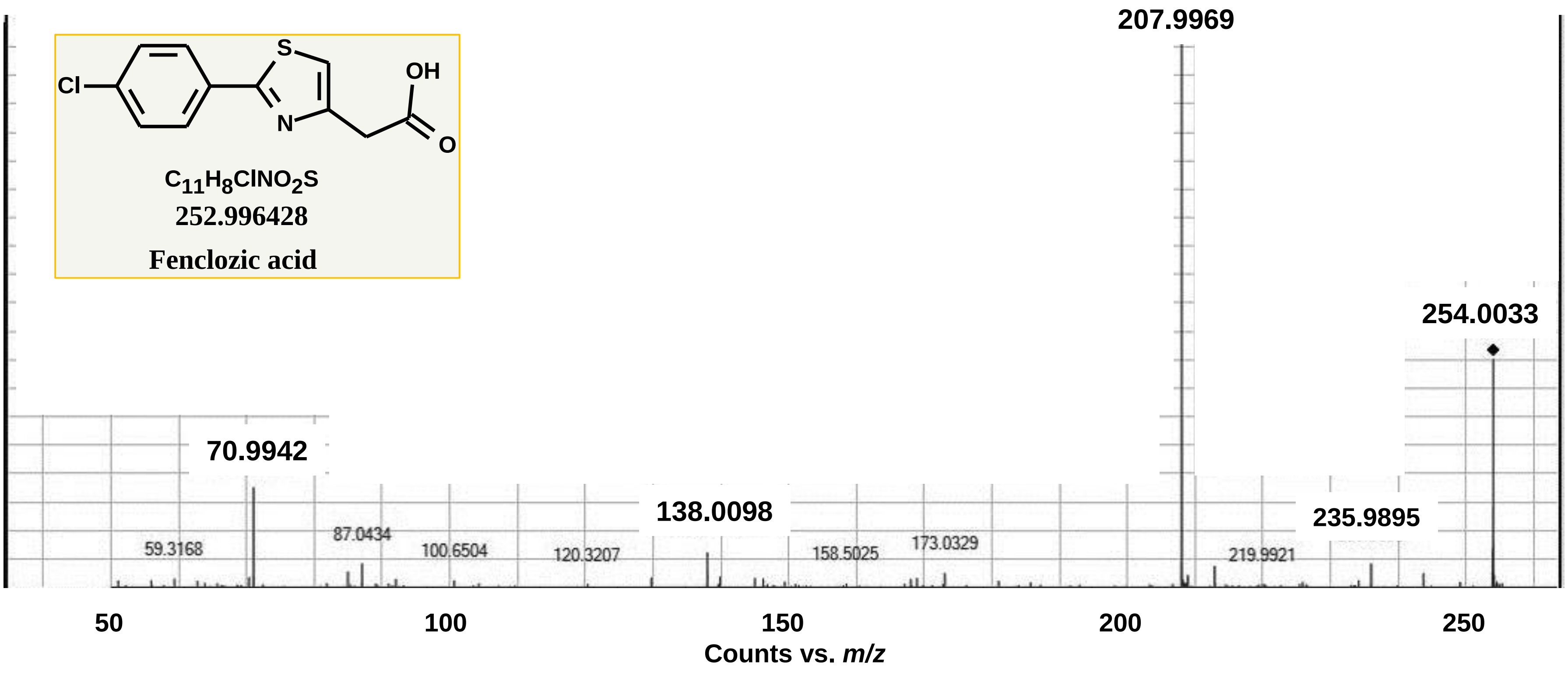

NB! In order to benefit from the chlorine isotope cluster in acquired MSMS mass spectra of all metabolites, the data were collected using a preset precursor ion mass window of ± 2 Da. The precursor ion mass was fixed onto the Cl-35 isotopolog which implies that the mass peak of the Cl-37 isotopolog was on the border of being included in the ion beam passing into the mass analyzer. Thus, the Cl-35/Cl-37 cluster in chlorine-containing fragment ions were indeed detected, albeit not always at the expected 3:1 ratio. This is seen in MSMS mass spectra of metabolites M1-M5, M8, M10-M14, M25, M26, M34, M35 and M37 in the following slides.

Figure S2. Fragment ion formation pathways of protonated fenclozic acid.

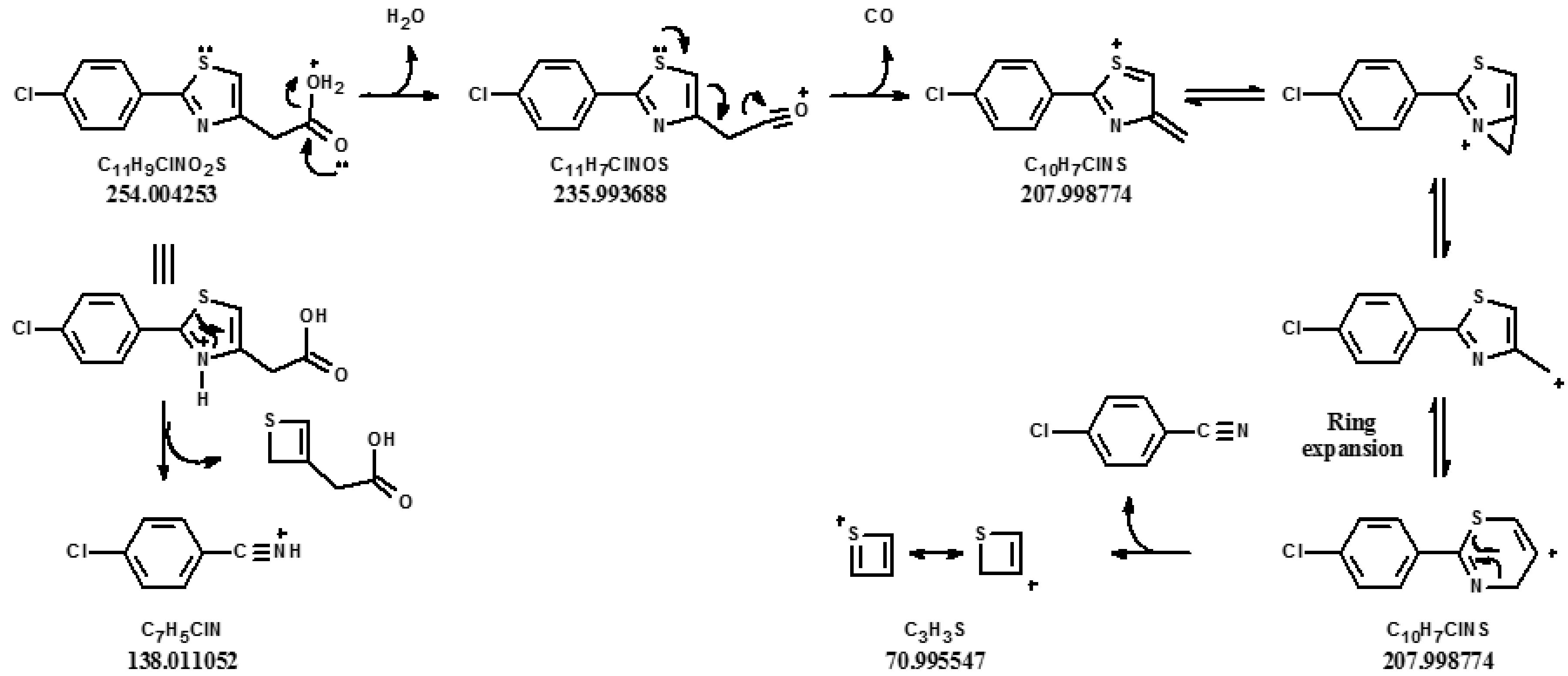

Figure S3. Metabolites of fenclozic acid seen in Bile.

Bile

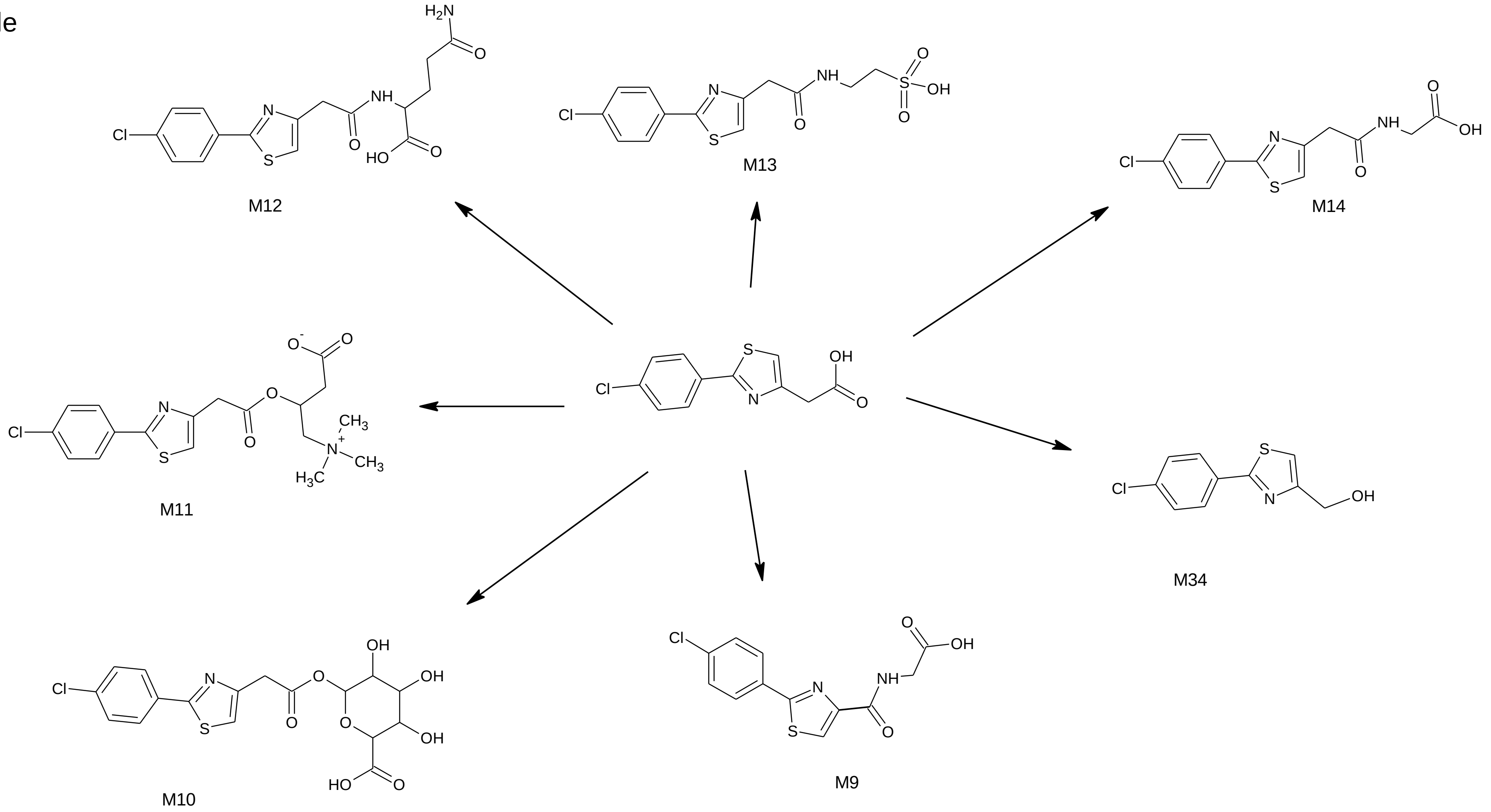

Figure S4. Metabolites of fenclozic acid seen in Liver extracts.

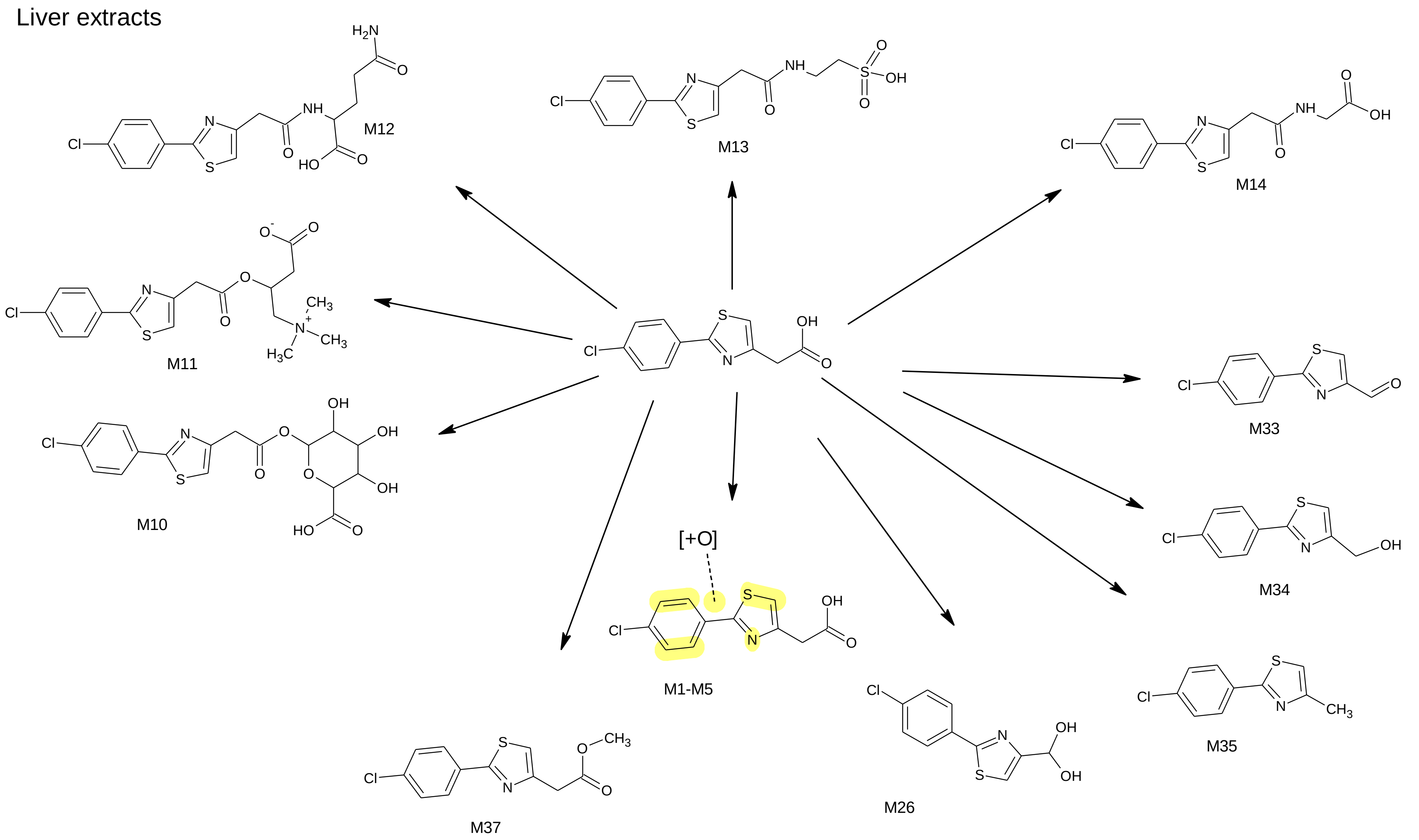

Figure S5. Metabolites of fenclozic acid seen in Feces extracts.

Feces extracts

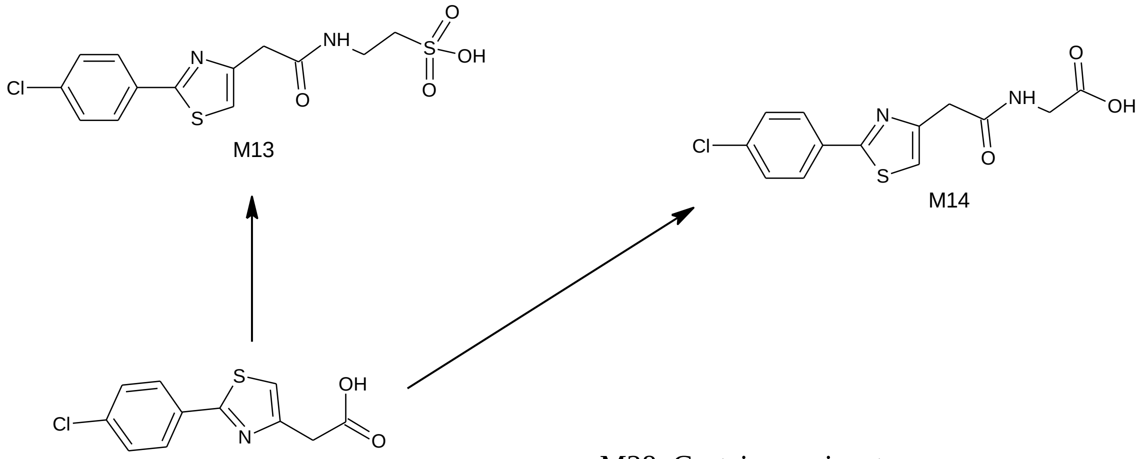

M28: Cysteine conjugate

Figure S6. Metabolites of fenclozic acid seen in Kidney extracts.

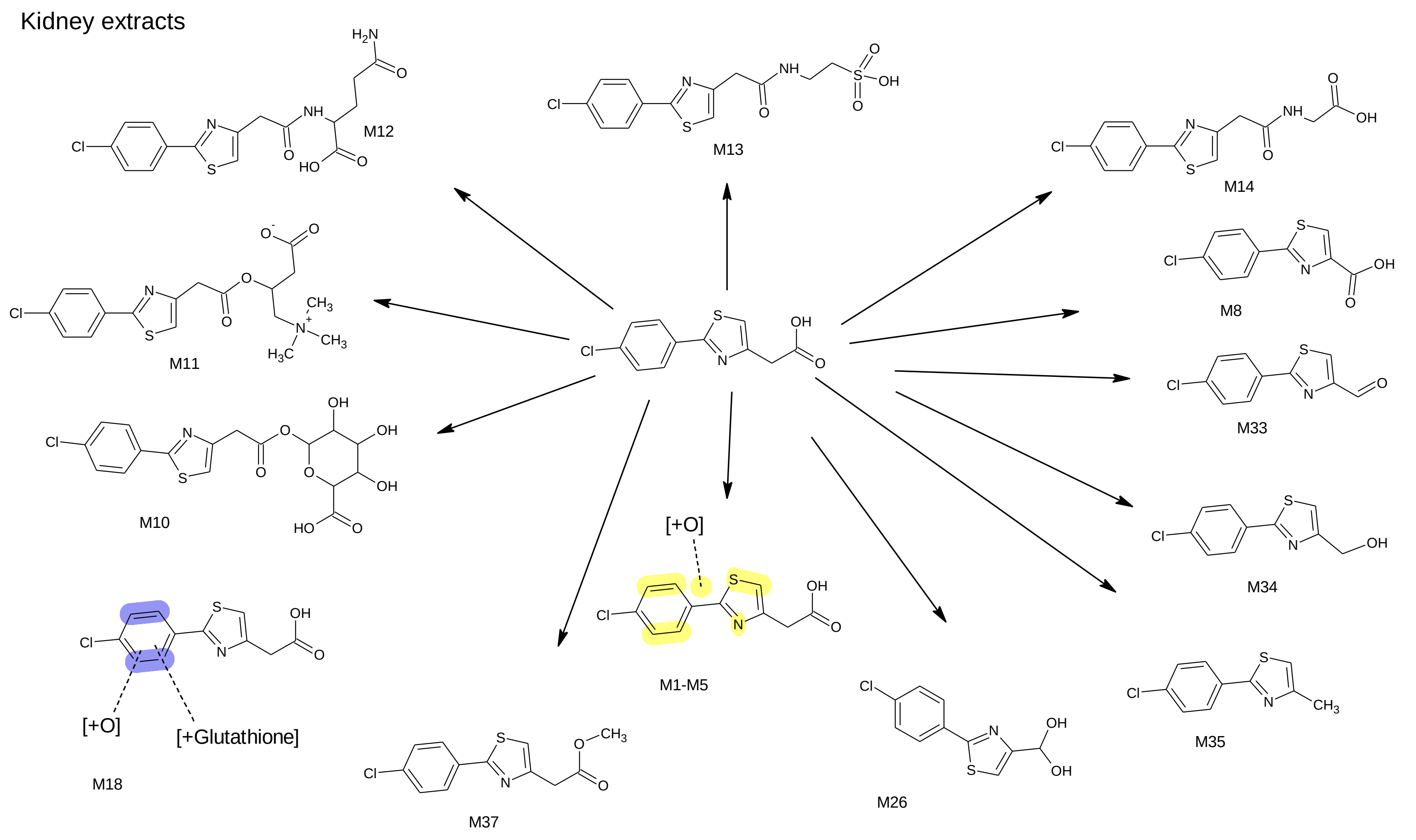

M28: Cysteinyl conjugate

M29: hydroxylation + glucuronidation = M20/M22/M23

Figure S7. Metabolites of fenclozic acid seen in Urine.

Urine

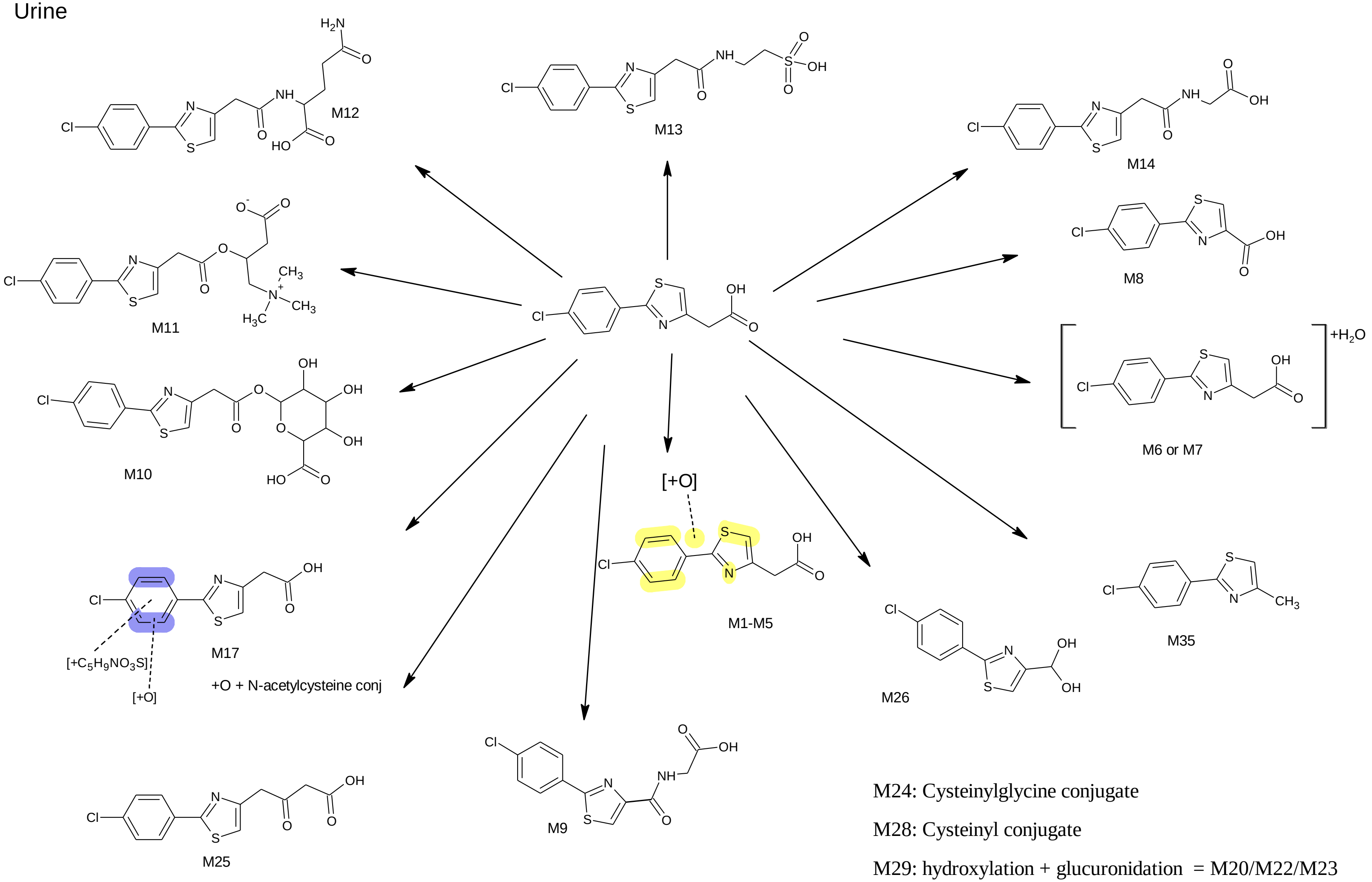

M24: Cysteinylglycine conjugate

M28: Cysteinyl conjugate

M29: hydroxylation + glucuronidation = M20/M22/M23

M30:N-acetylcysteine conjugate

M31: N-acetylcysteinyl conjugate

Figure S8. Monohydroxylated FAH^+, one example of five isomers (M1-M5)

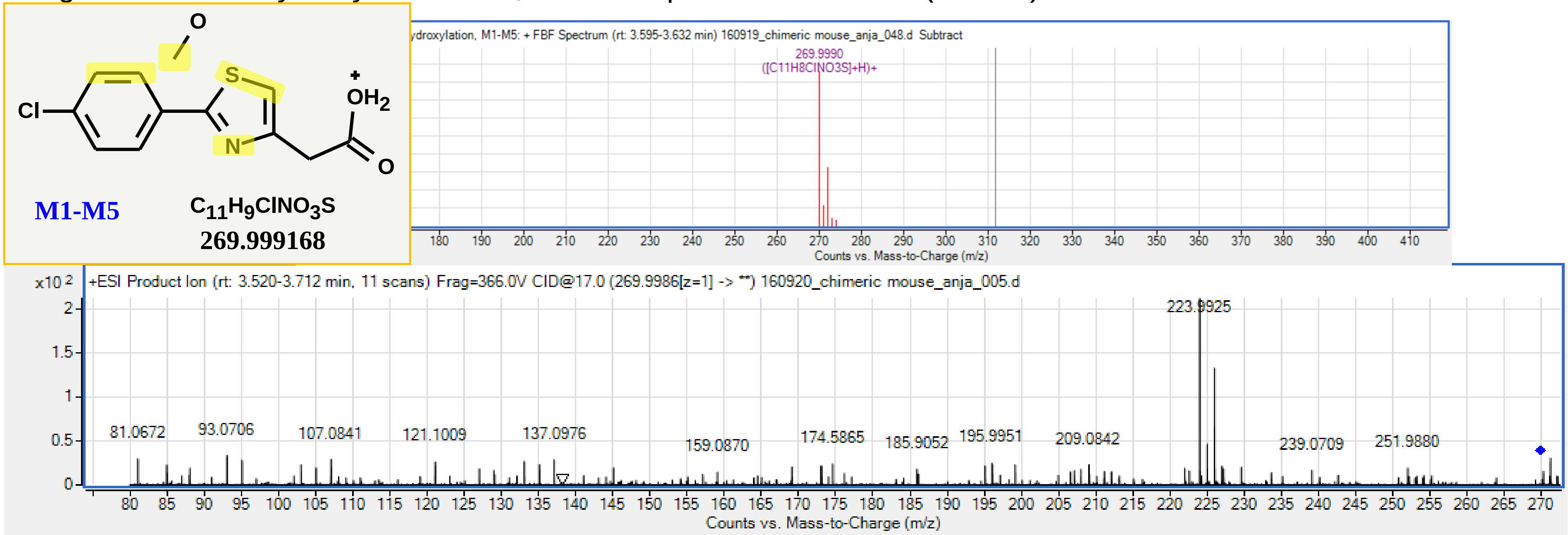

Figure S9. Hydrated FAH^+, one example of two isomers (M6, M7)

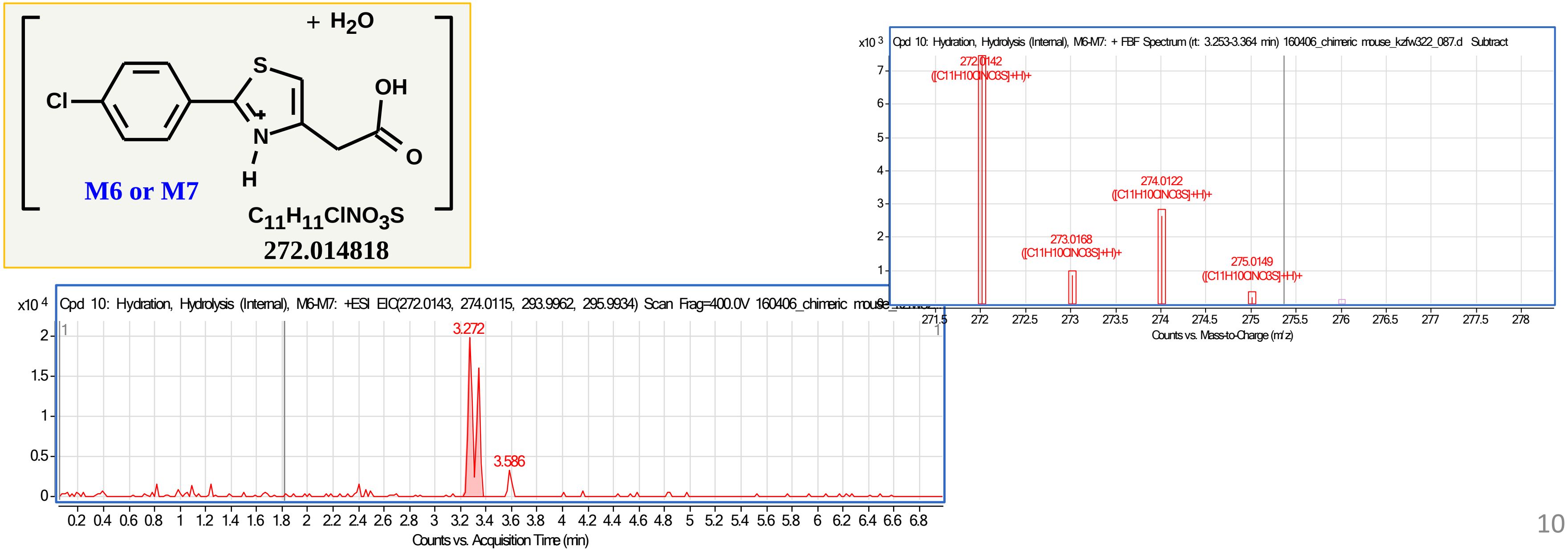

Figure S10. Chain-shortened acid FAH+ (M8)

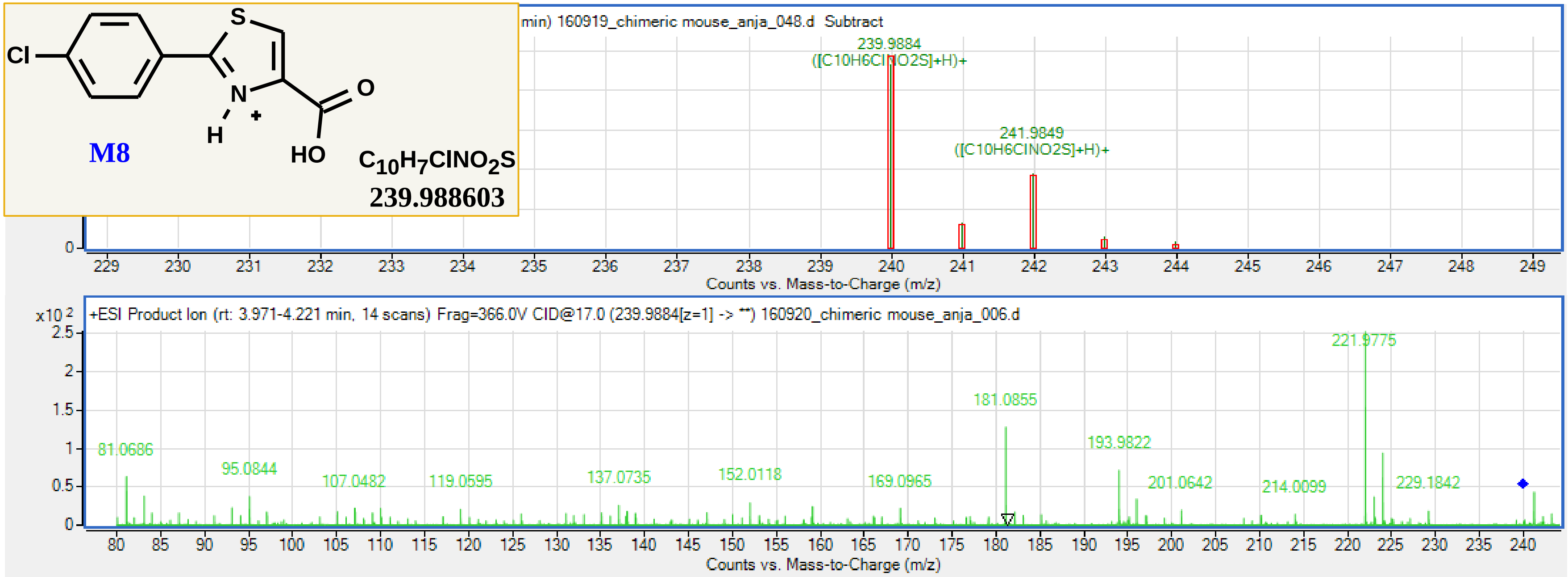

Figure S11. Decarboxylated FAH+ glycine conjugate (M9)

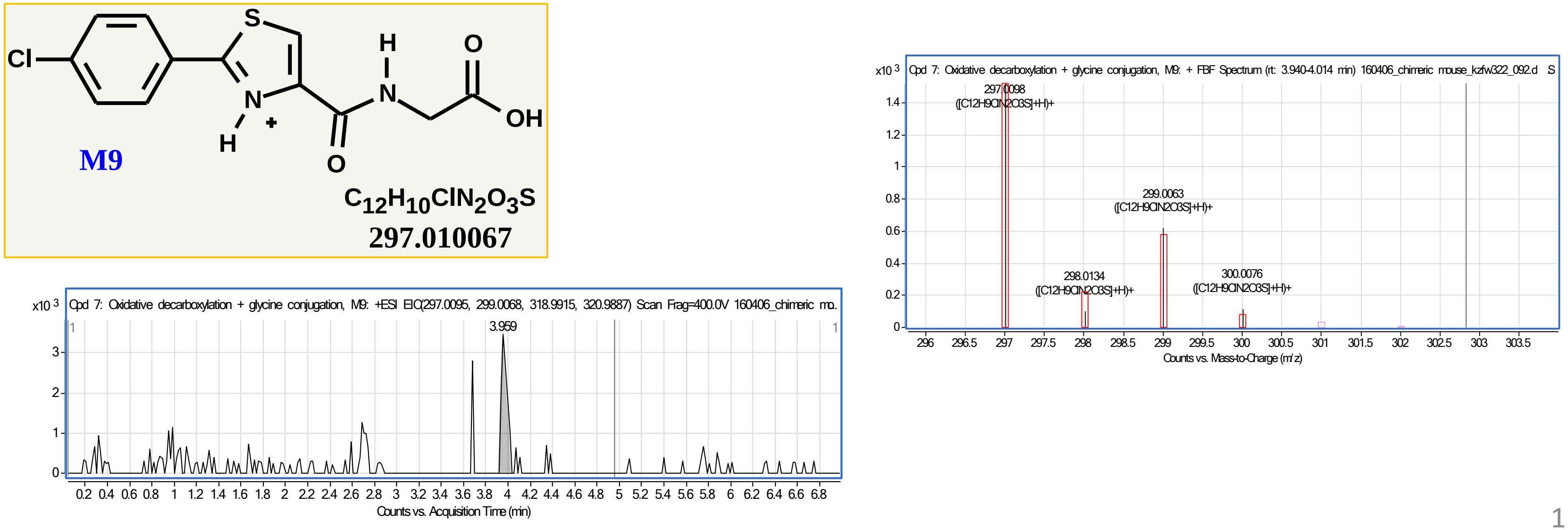

Figure S12. FAH+ acyl glucuronide (M10)

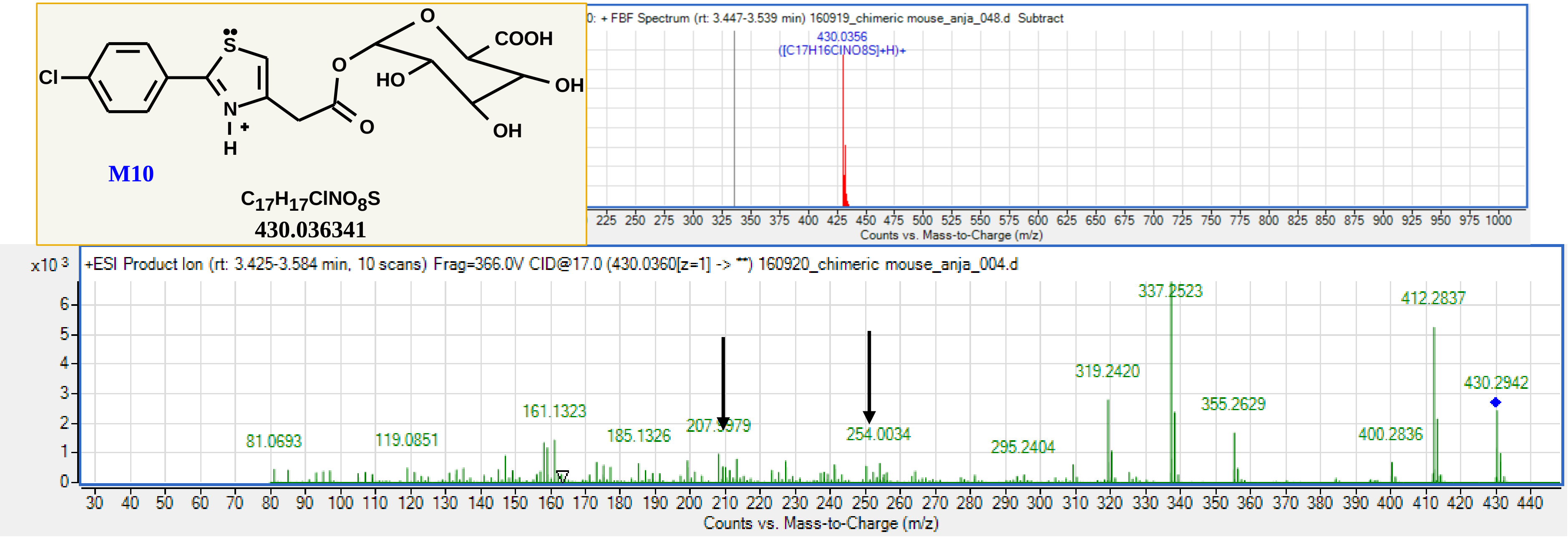

Figure S13. Carnitine conjugated FAH+ (M11)

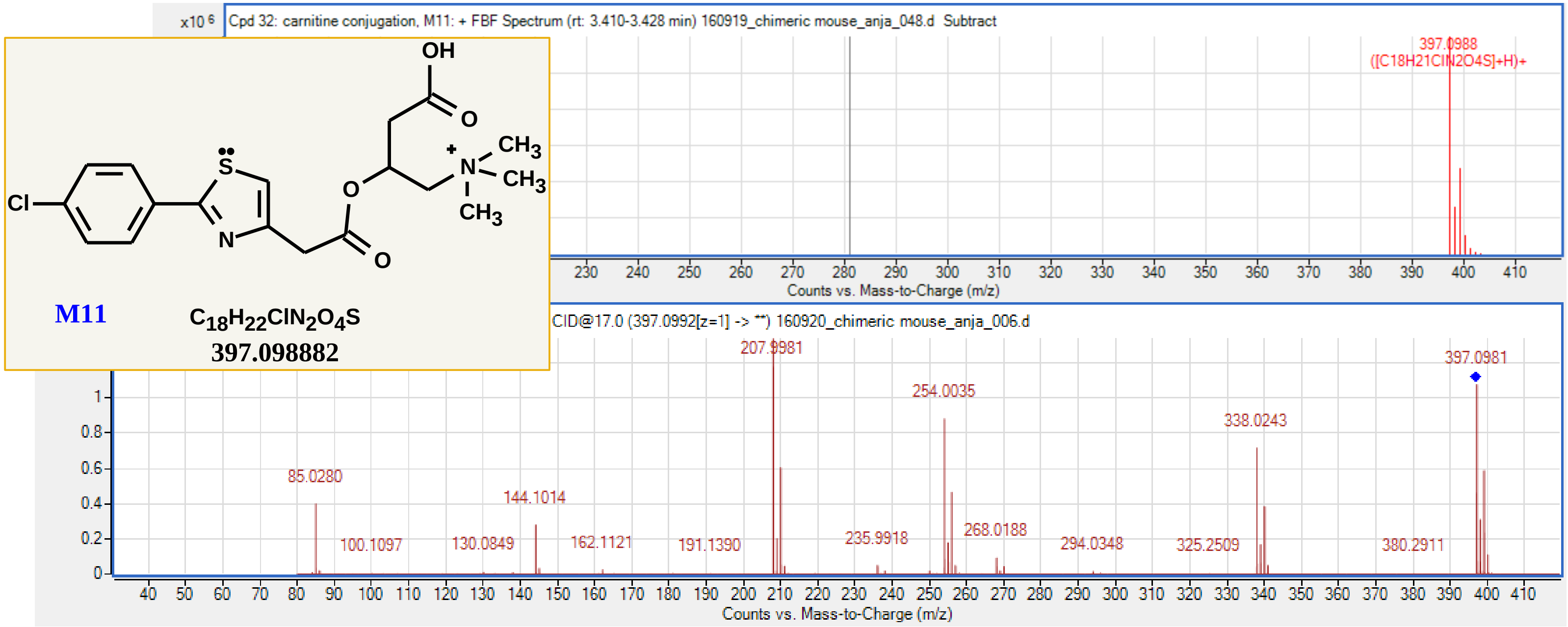

Figure S14. Glutamic acid conjugated FAH^+ (M12)

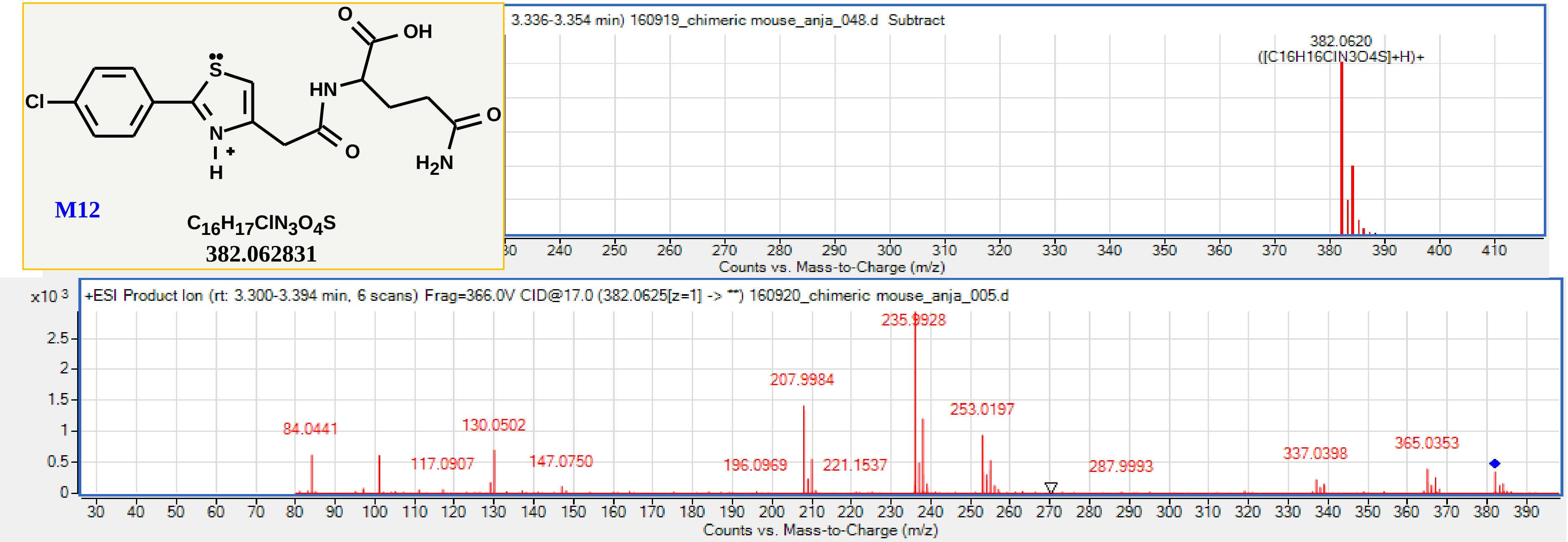

Figure S15. Taurine conjugated FAH^+ (M13)

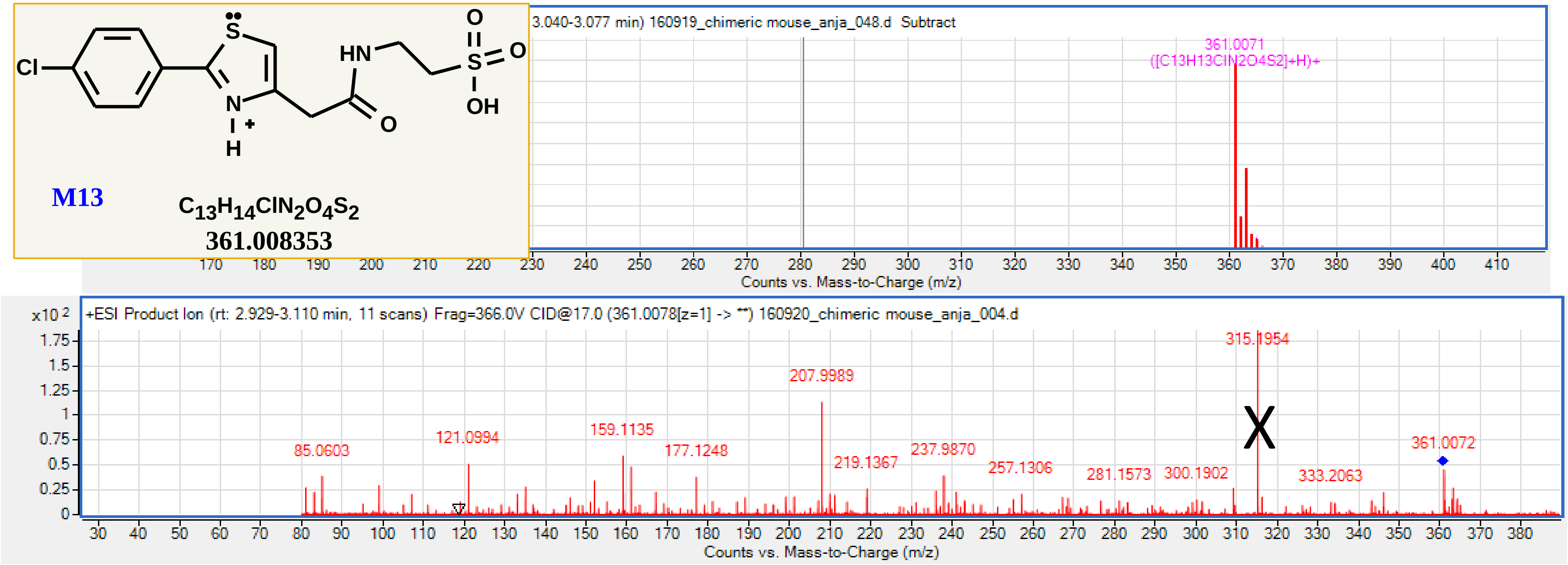

Figure S16. Glycine conjugated FAH+ (M14)

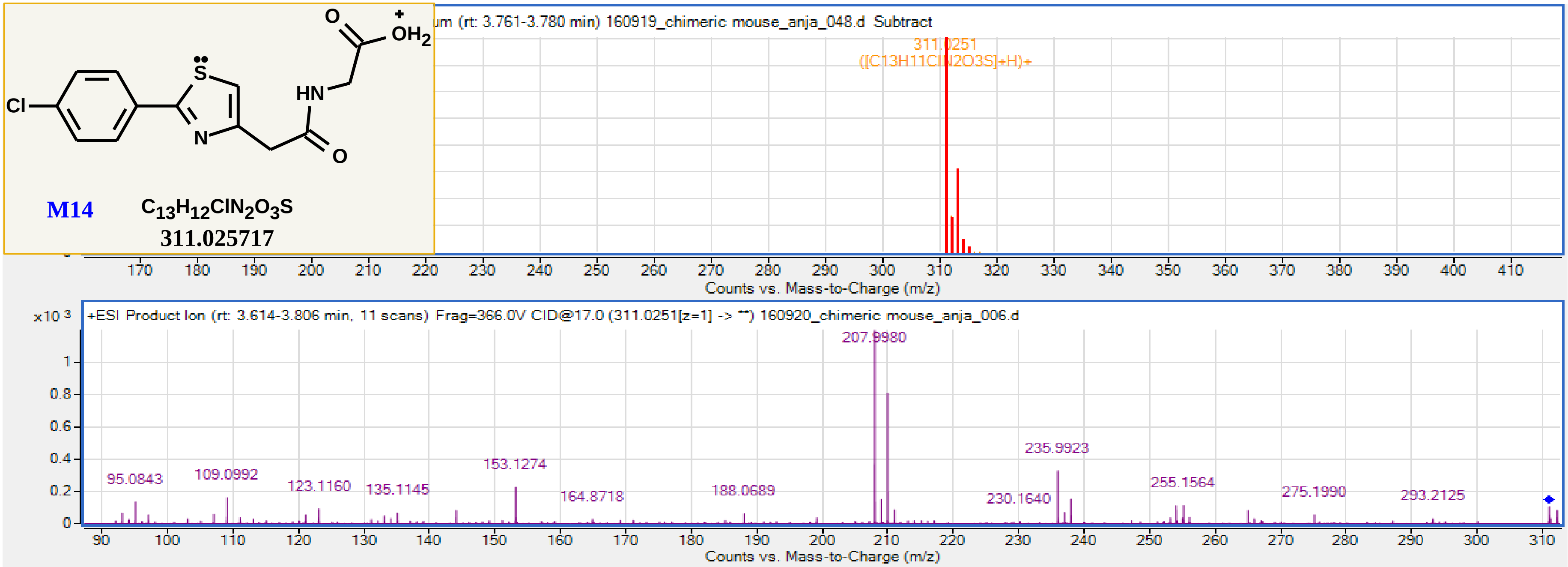

Figure S17. FAH+ + O + NAc-Cys-SH (M17)

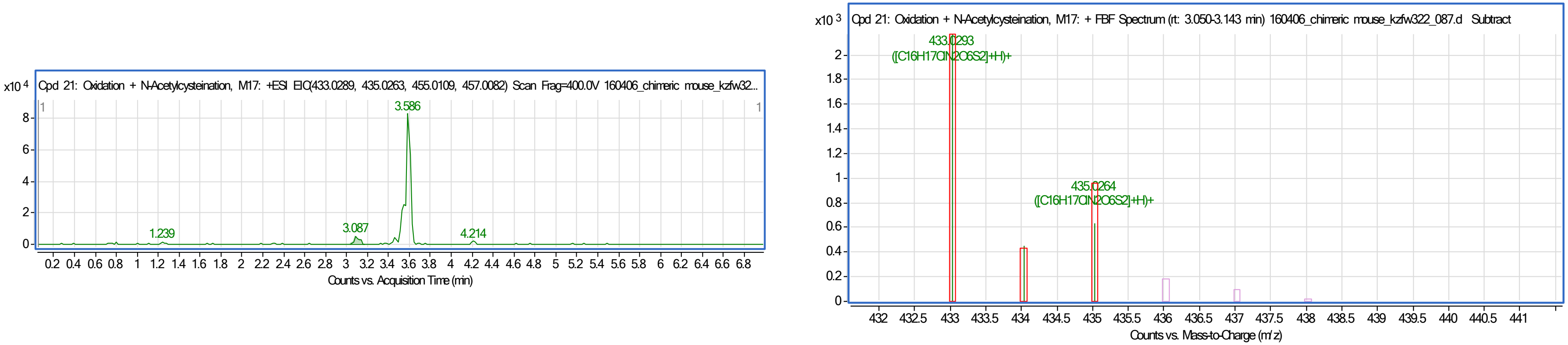

Figure S18. FAH^{+} + HS-CysGly – 2 H (M24)

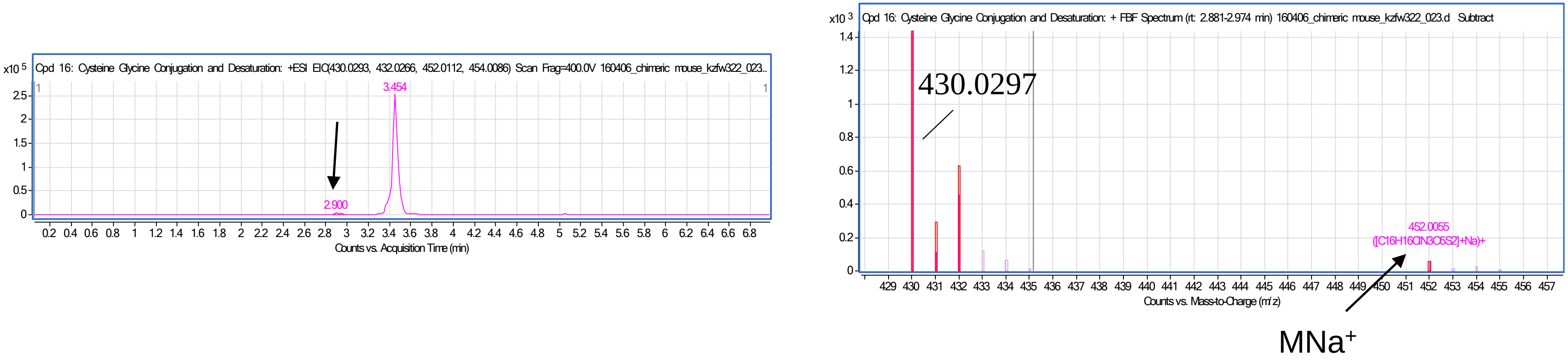

Figure S19. Chain elongated FAH^{+} (M25)

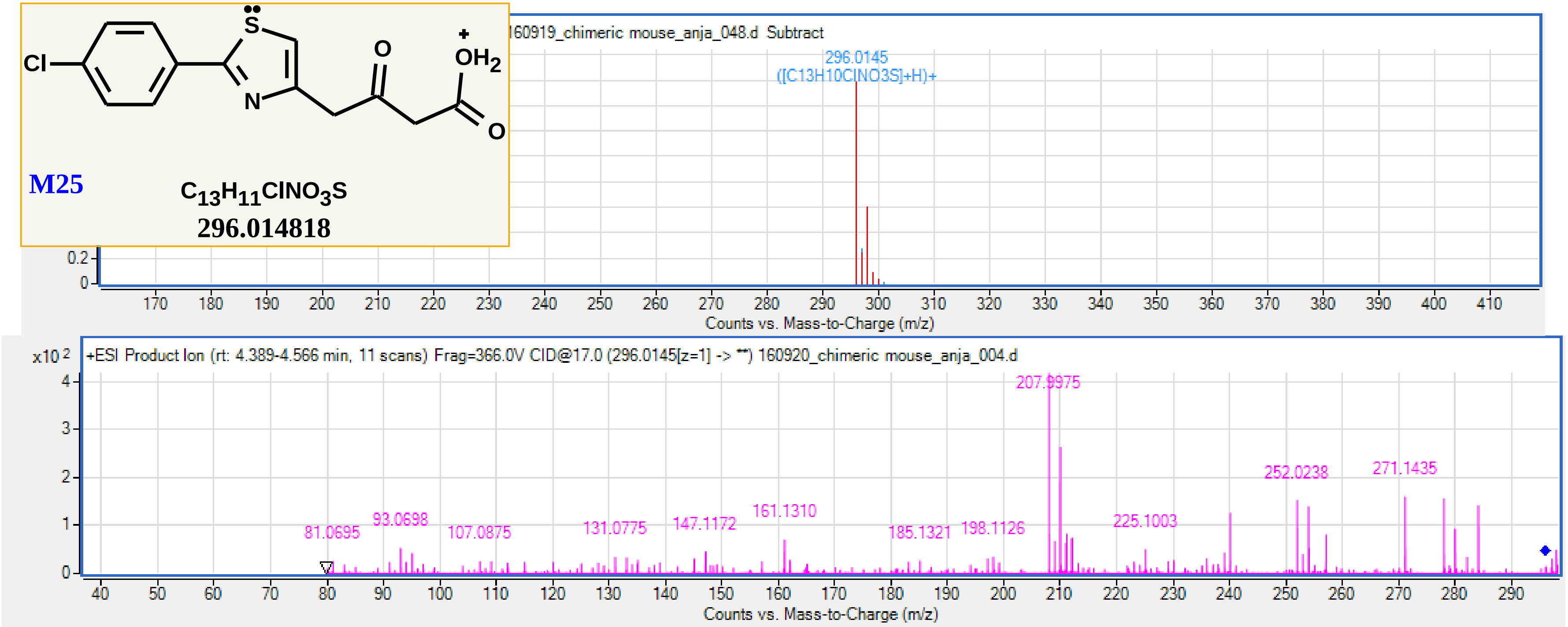

Figure S20. Decarboxylated FAH$^+$ hydrated aldehyde (M26)

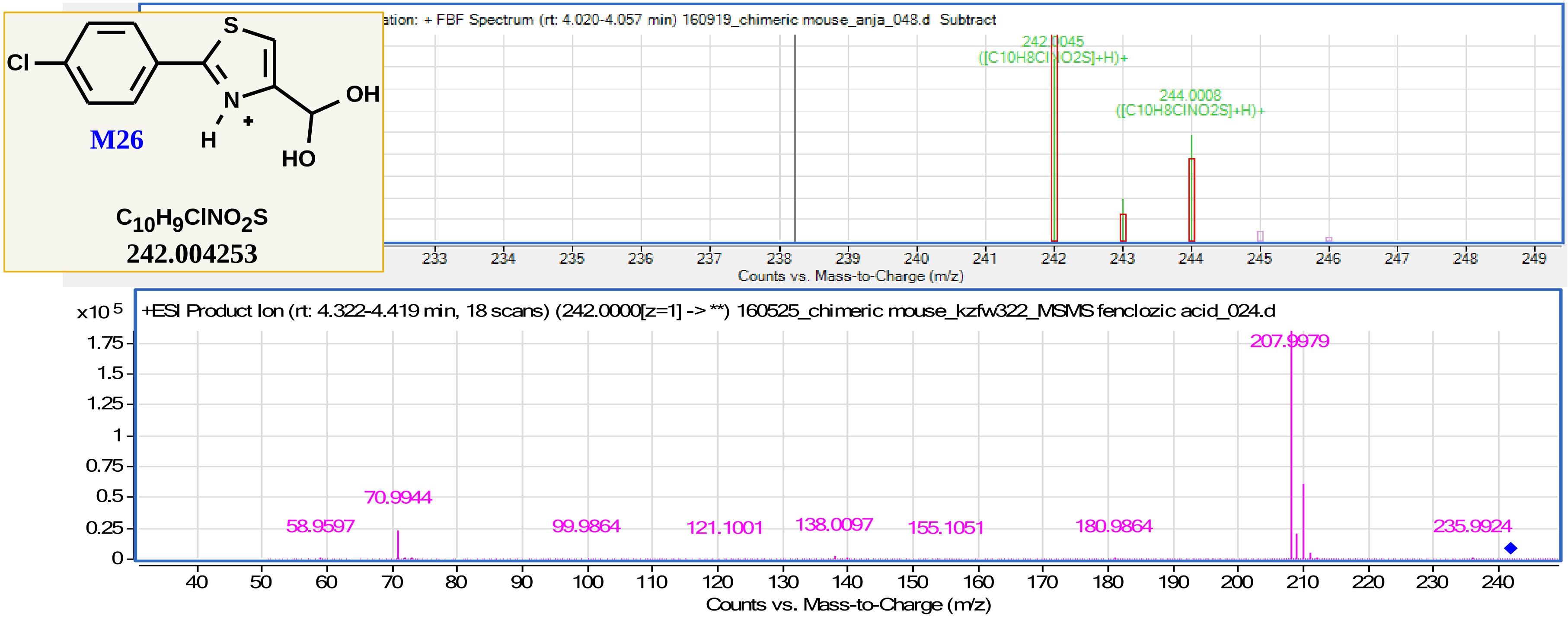

Figure S21. FAH$^+$ + Cys-SH (M27)

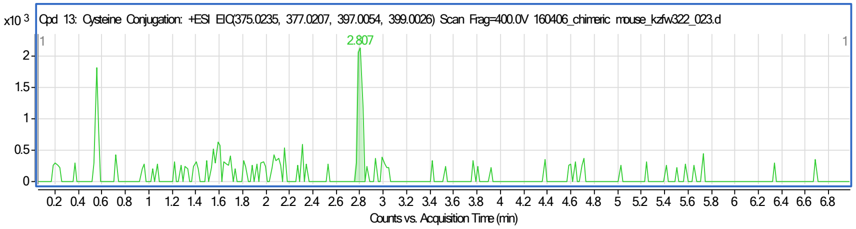

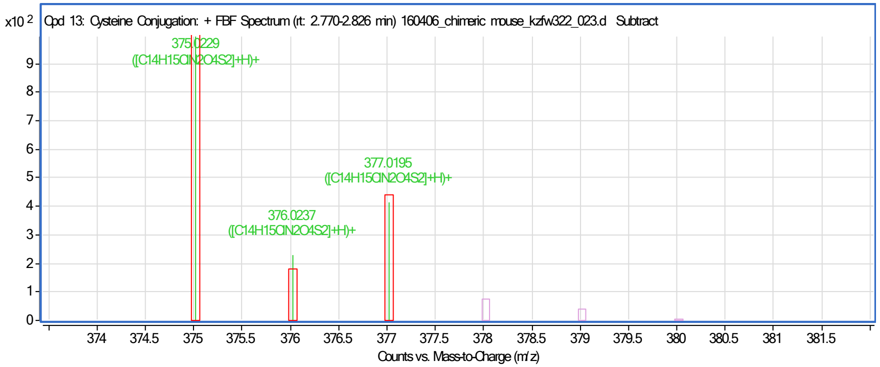

Figure S22. FAH$^+$ + Cys-SH – 2 H (M28)

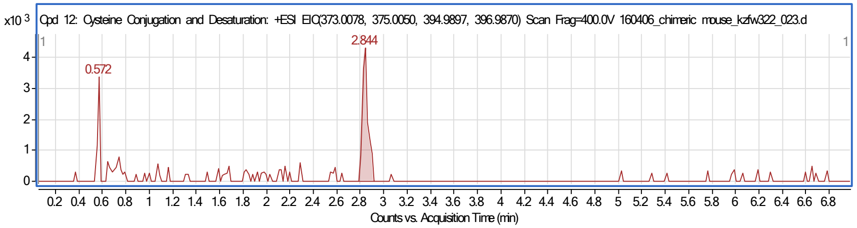

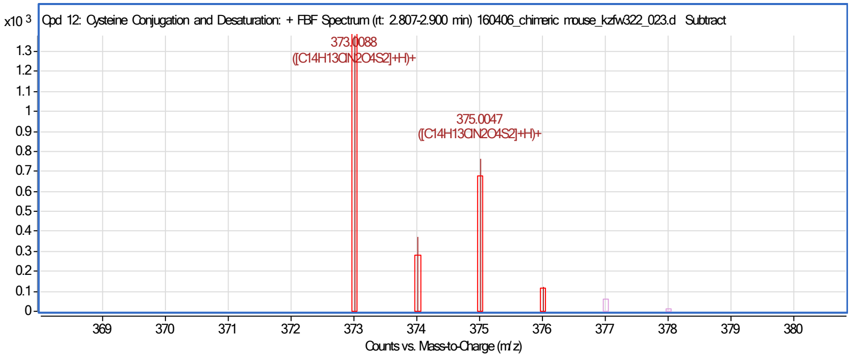

Figure S23. Hydroxylated FAH$^+$ glucuronide (M29) (Previously M20, M22 or M23; *Martin 2014, Pickup 2017*)

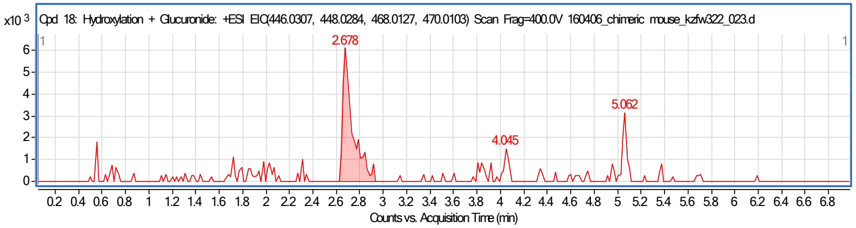

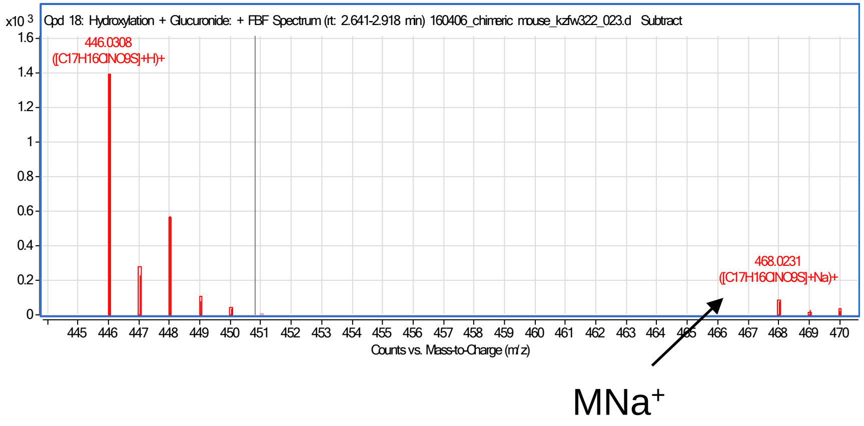

Figure S24. FAH$^+$ + NAc-Cys-SH (M30)

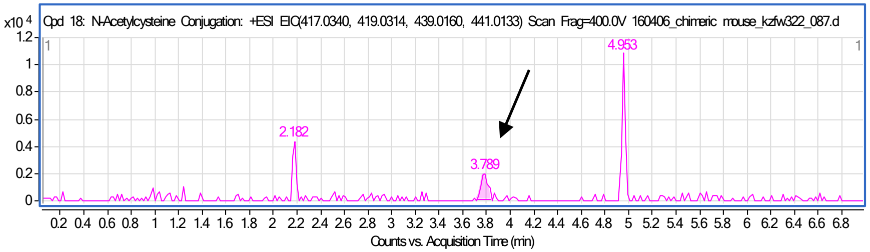

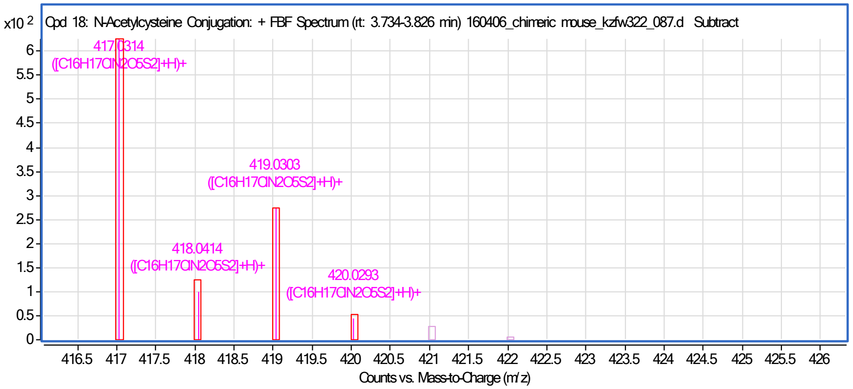

Figure S25. FAH$^+$ + NAc-Cys-SH - 2 H (M31)

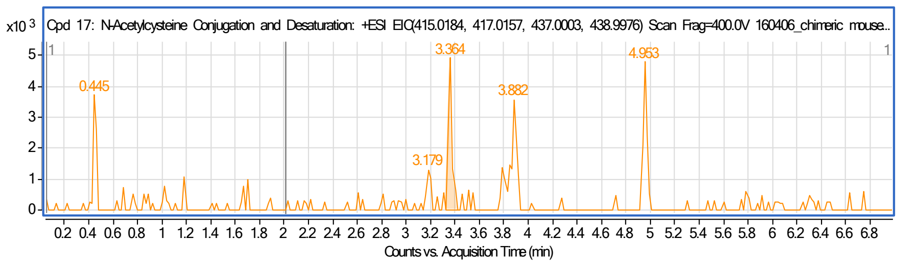

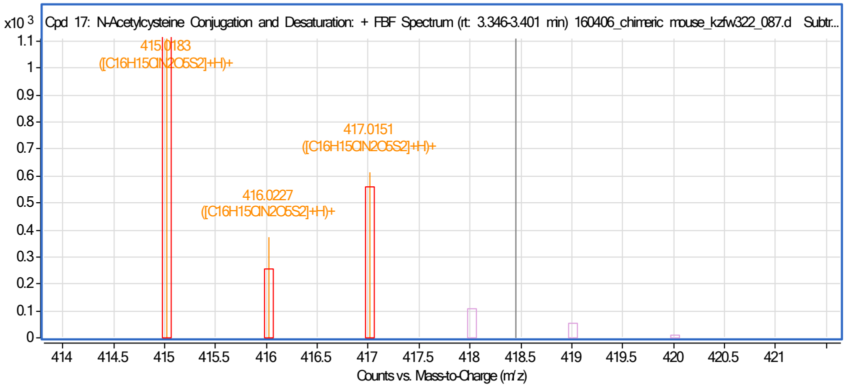

Figure S26. Decarboxylated FAH$^+$ aldehyde (M33)

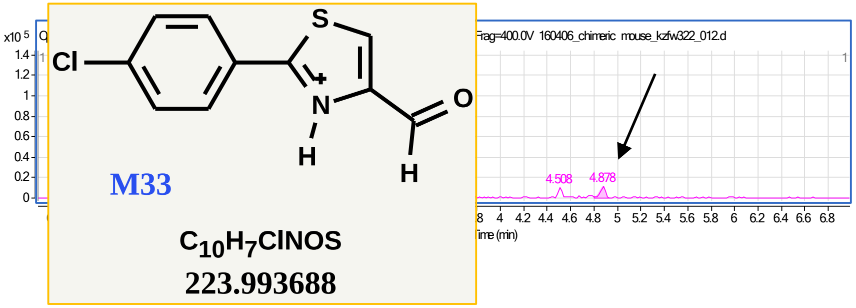

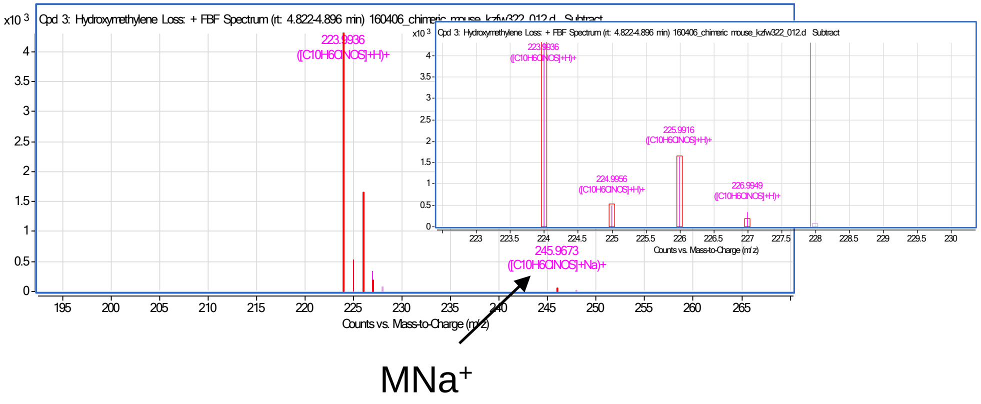

Figure S27. Decarboxylated and hydroxylated FAH^{+} (M34)

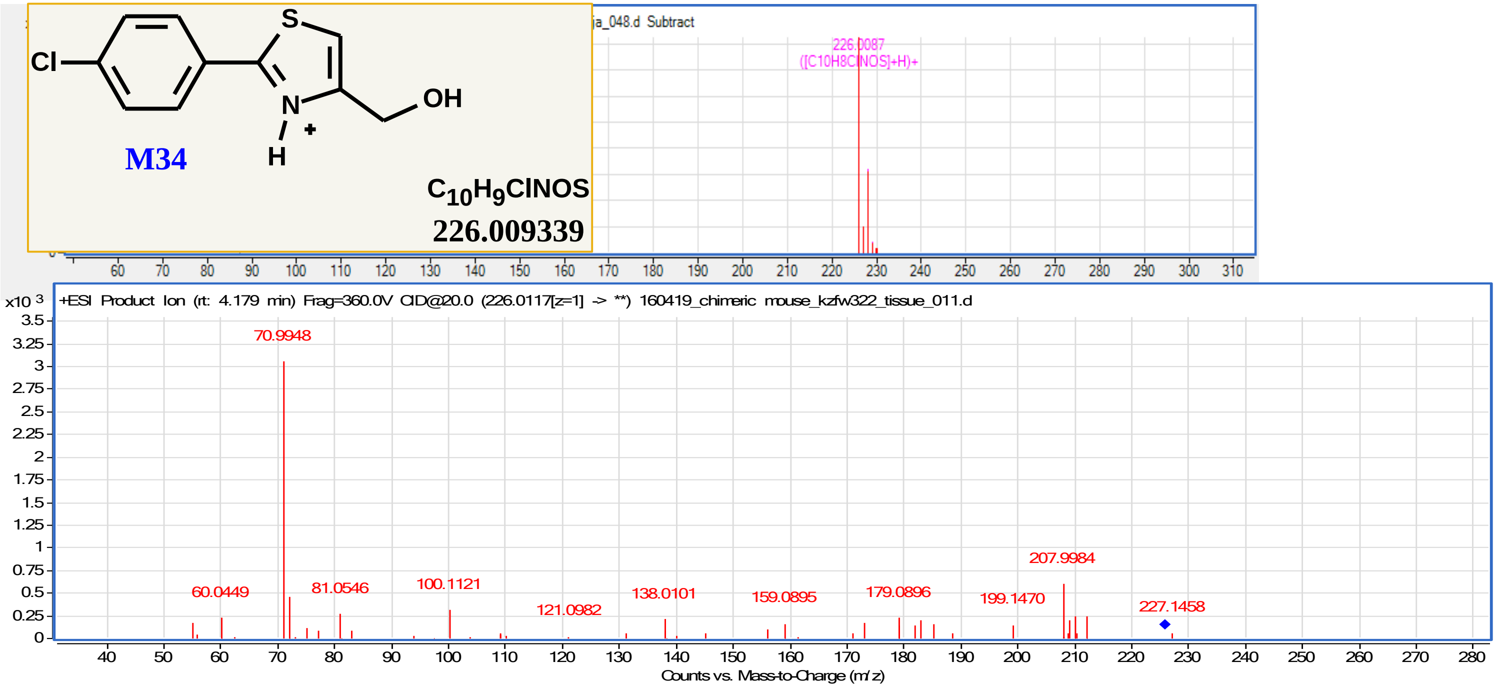

Figure S28. Decarboxylated FAH^{+} (M35)

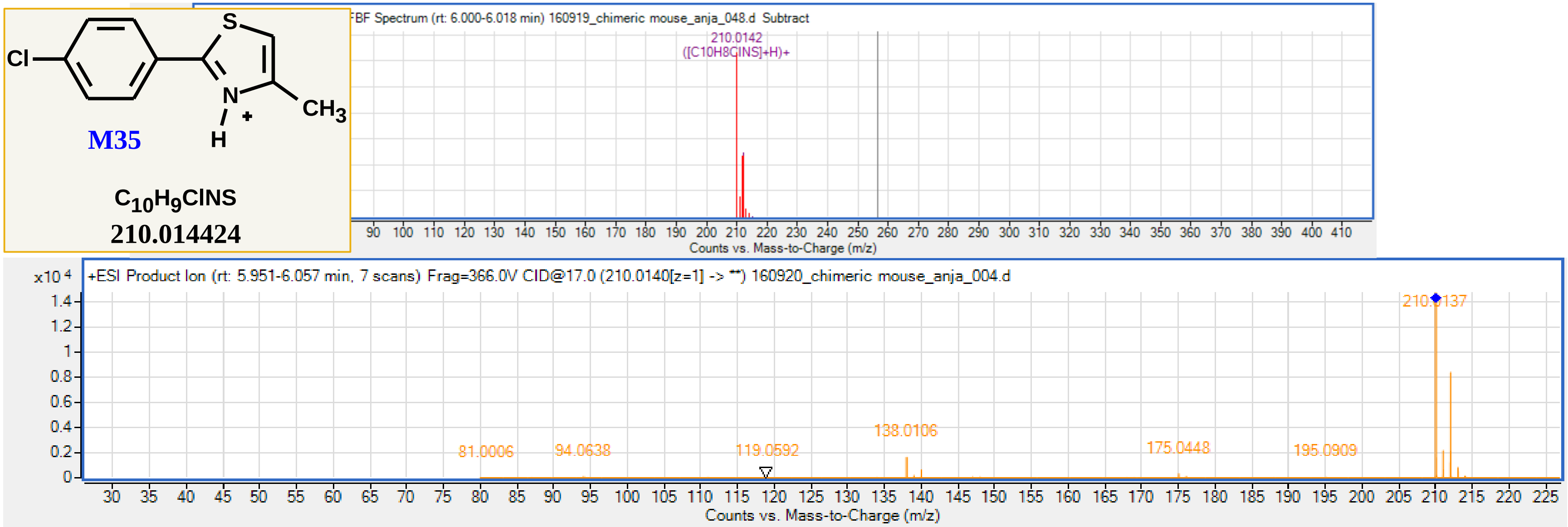

Figure S29. Trioxygenated FAH^{+} (M36)

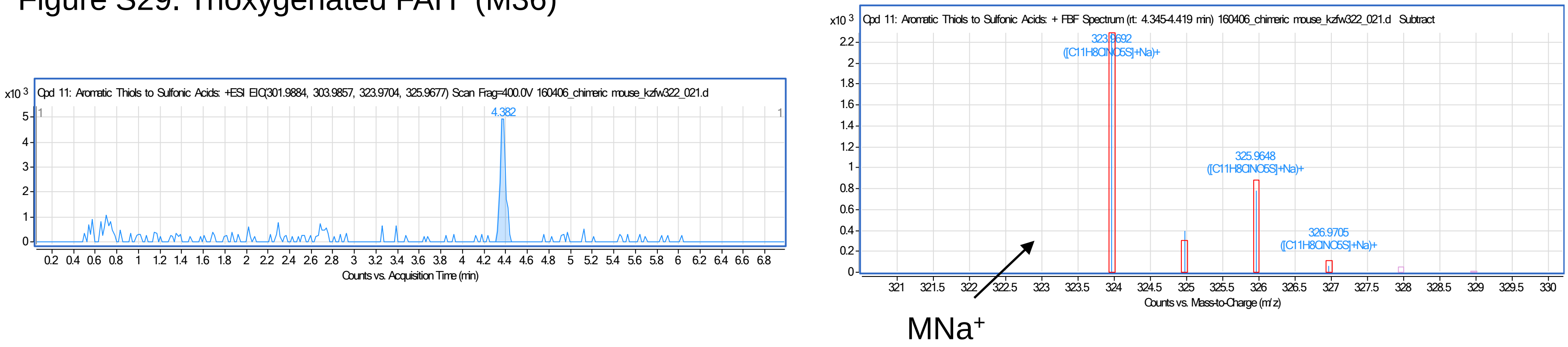

Figure S30. FAH^{+} methyl ester (M37)

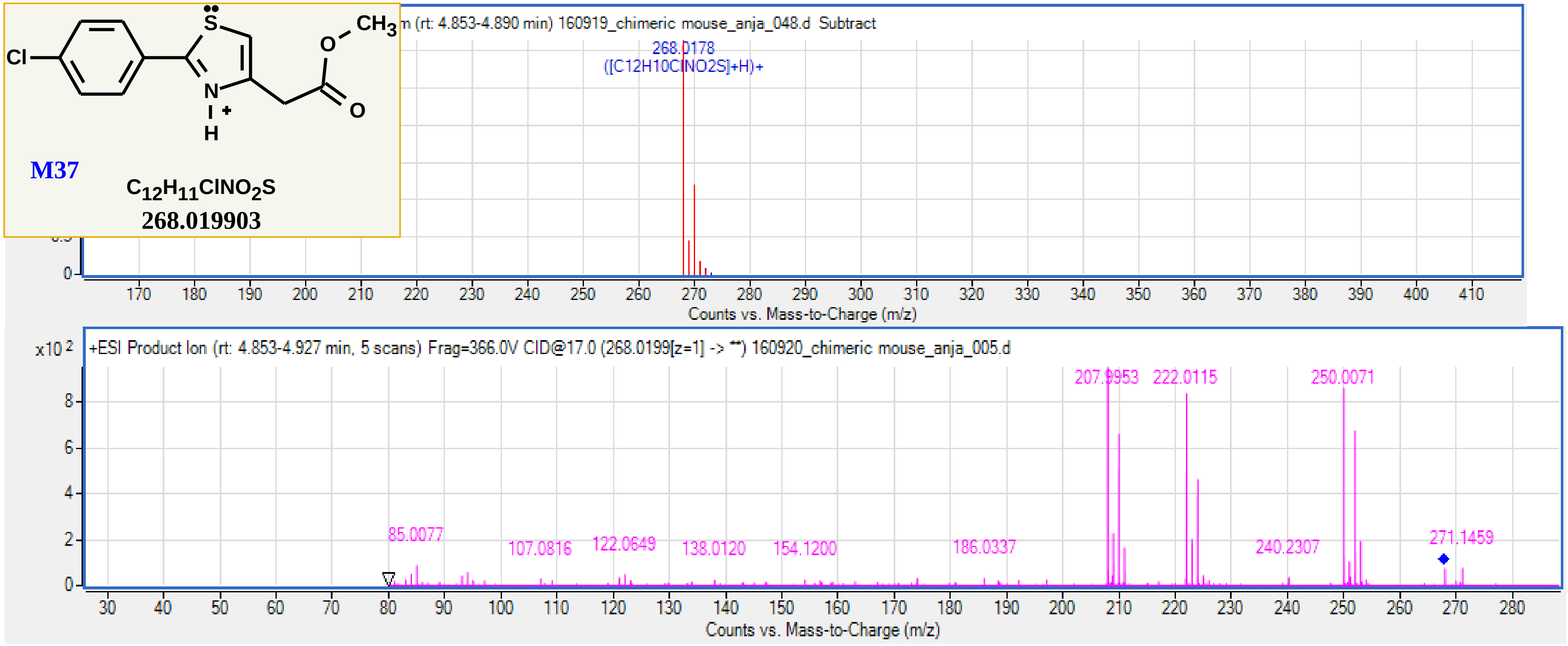

Supplement: Supplementary file 1 — Supplementary material 1 (PDF 739 KB) [file 204_2018_2274_MOESM1_ESM.pdf]
